# The Transposon *Galileo* Generates Natural Chromosomal Inversions in Drosophila by Ectopic Recombination

**DOI:** 10.1371/journal.pone.0007883

**Published:** 2009-11-18

**Authors:** Alejandra Delprat, Bàrbara Negre, Marta Puig, Alfredo Ruiz

**Affiliations:** Departament de Genètica i de Microbiologia, Universitat Autònoma de Barcelona, Bellaterra (Barcelona), Spain; American Museum of Natural History, United States of America

## Abstract

**Background:**

Transposable elements (TEs) are responsible for the generation of chromosomal inversions in several groups of organisms. However, in Drosophila and other Dipterans, where inversions are abundant both as intraspecific polymorphisms and interspecific fixed differences, the evidence for a role of TEs is scarce. Previous work revealed that the transposon *Galileo* was involved in the generation of two polymorphic inversions of *Drosophila buzzatii*.

**Methodology/Principal Findings:**

To assess the impact of TEs in *Drosophila* chromosomal evolution and shed light on the mechanism involved, we isolated and sequenced the two breakpoints of another widespread polymorphic inversion from *D. buzzatii*, *2z* ^3^. In the non inverted chromosome, the *2z* ^3^ distal breakpoint was located between genes *CG2046* and *CG10326* whereas the proximal breakpoint lies between two novel genes that we have named *Dlh* and *Mdp*. In the inverted chromosome, the analysis of the breakpoint sequences revealed relatively large insertions (2,870-bp and 4,786-bp long) including two copies of the transposon *Galileo* (subfamily *Newton*), one at each breakpoint, plus several other TEs. The two *Galileo* copies: (i) are inserted in opposite orientation; (ii) present exchanged target site duplications; and (iii) are both chimeric.

**Conclusions/Significance:**

Our observations provide the best evidence gathered so far for the role of TEs in the generation of Drosophila inversions. In addition, they show unequivocally that ectopic recombination is the causative mechanism. The fact that the three polymorphic *D. buzzatii* inversions investigated so far were generated by the same transposon family is remarkable and is conceivably due to *Galileo'*s unusual structure and current (or recent) transpositional activity.

## Introduction

A sizable portion of eukaryotic and prokaryotic genomes is composed of transposable elements (TEs) with the potential to cause chromosomal rearrangements such as inversions, translocations and duplications [1]–[3]. These rearrangements however may be generated also by other processes that do not involve TEs (see below). Thus, the actual contribution of TEs to the evolutionary reorganization of genomes is unclear. One of the most frequent and widespread types of chromosomal rearrangements during evolution are inversions, which alter gene order often without changing total gene content [4]. Inversions are remarkably abundant in the genus Drosophila, both as intraspecific polymorphisms and as interspecific fixed differences [5], [6] and increasing evidence point to their prevalence in many other species, e.g. humans [7]–[11].

TEs can generate chromosomal inversions by intrachromosomal homologous recombination between two copies of the same TE family arranged in opposite orientation [12]. This mechanism is known as TE-mediated ectopic recombination or nonallelic homologous recombination (NAHR). TEs can also induce inversions as well as other types of rearrangements when two ends coming from different TE copies participate together in an aberrant transposition event. The outcome depends on the location and orientation of the two cooperating TE copies in the parental chromosome and the chromosomal site where they insert (Figure S1). If the two copies are located in sister chromatids or homologous chromosomes, the process is referred to as hybrid element insertion [13]–[15]. When the two copies are located at neighboring sites on the same chromatid, the mechanism is known as reversed ends transposition [16], [17]. Inversions can be also generated by two other mechanisms not involving TEs. One such mechanism is chromosomal breakage and repair by non-homologous end-joining (NHEJ). Double strand breaks (DSBs) are produced in many ways in all cells and the machinery to deal with these lesions is conserved from yeasts to vertebrates [18], [19]. When two or more DSBs occur simultaneously, repair by NHEJ may produce gross rearrangements if the joining takes place between previously unlinked DNA molecules [20]. Finally, inversions may result from ectopic recombination between other repeated sequences besides TEs, such as tRNA genes [21] or segmental duplications (SDs) [7], [8].

TE-mediated ectopic recombination has generated natural chromosomal inversions in bacteria [22]–[27] and some lineages have experienced an striking degree of rearrangement caused by this process [28]–[31]. Likewise, *Ty*-recombination mediated deletions, duplications, inversions and translocations have been found to occur in yeast [12], [32]–[34]. In mammals, long and short interspersed elements (LINEs and SINEs, respectively) have been implicated in the generation by ectopic recombination of 50 inversions fixed between humans and chimpanzees [35], [36]. In Drosophila, the evidence for the implication of TEs in the generation of inversions is limited. Two *D. buzzatii* polymorphic inversions, *2j* and *2q* ^7^, were seemingly generated by ectopic recombination between copies of the transposon *Galileo* [37], [38]. In *D. pseudoobscura*, the polymorphic inversion Arrowhead and a number of fixed inversions have been also generated by ectopic recombination between 128-bp and 315-bp repeats, yet the nature of these repeats is obscure [39]. Inversion *In(4)a* of *D. americana* has been found to be flanked by copies of a new transposon and was likely generated by an intrachromosomal exchange between these repeats [40]. TEs have been found also at the breakpoints of two *Anopheles gambiae* inversions, *2Rd'* and *2La*, but the implication of these TEs in the origin of the inversions is circumstantial [41], [42].

Chromosomal breakage and repair by NHEJ is also a common mechanism for the generation of chromosomal inversions. This process may generate duplications flanking the inverted segment when one or both DSBs occur in a staggered manner [43]. In Drosophila, this process has been responsible for most of the inversions fixed between *D. melanogaster* and *D. yakuba* [43] as well as three *D. melanogaster* polymorphic inversions [44]–[46]. In addition, this mechanism likely generated several inversions fixed in other lineages where TEs were not detected at the breakpoints or when present were not involved in the origin of the inversion [47]–[50]. SDs represent a significant fraction of mammalian genomes and ectopic recombination between SDs seems to be a common mechanism inducing chromosomal inversions in these genomes. Six of the nine large pericentric inversion differences between the human and chimpanzee genomes have been associated with SDs [51] and there is a significant SD enrichment at the sites of breakpoints which occurred during primate evolution [52]–[58] although it is not clear whether ectopic recombination is always the cause for the co-location of SDs and breakpoints. Ectopic recombination between SDs is also responsible for the generation of chromosomal inversions in other groups, e.g insects [59].

The transposon *Galileo* was discovered in *D. buzzatii* and tentatively classified (along with two related elements named *Newton* and *Kepler*) as a *Foldback*-like element because of its long, internally repetitive, terminal inverted repeats (TIRs) and lack of coding capacity [60], [61]. We have recently shown that *Galileo* is a cut-and-paste transposon belonging to the *P* superfamily that is present in six of the 12 recently sequenced Drosophila genomes [62]. *Galileo*, *Newton* and *Kepler* show a high degree of nucleotide similarity (including the most terminal 40 bp that are almost identical) and produce 7-bp target site duplications (TSDs) with the same consensus sequence, GTAGTAC, which suggests that they are mobilized by the same transposase [61]. They should be considered only as different subfamilies of *Galileo* in the genome of *D. buzzatii* and will be denoted hereafter as *GalileoG*, *GalileoN* and *GalileoK*, respectively.

In order to increase our understanding of the mechanisms underlying the generation of Drosophila inversions in nature and test for an implication of transposable elements, here we isolated and characterized the breakpoints of another *D. buzzatii* polymorphic inversion, *2z* ^3^. This inversion arose on a chromosome carrying the *2j* inversion, giving rise to arrangement *2jz* ^3^. The *2z* ^3^ segment encompasses about one third of chromosome 2 (∼11 Mb) and overlaps the *2j* segment so that the two inversions can not be separated by recombination [63] Thus, three chromosome 2 arrangements are commonly found in *D. buzzatii* natural populations, *2 standard* (*2st*), *2j* and *2jz* ^3^. Arrangement *2jz* ^3^ has a wide geographical distribution being present in natural populations of Argentina, Southern Brazil, Chile and the Old World [64], [65]. In 18 Argentina populations where arrangement *2jz* ^3^ is present, its relative frequencies range from 0.5 to 31.5% with an average of ∼8% [65]. We choose to study this inversion in part because its proximal breakpoint was located at chromosomal band 2F1c [63] very near the site (2F1c-e) where the *proboscipedia*-*Ultrabithorax* portion of the Hox gene complex has been localized [66], [67]. We seek to determine the precise distance from the inversion breakpoint to the Hox genes and find out whether these genes were affected in any way by the inversion. The results show that copies of the transposon *GalileoN* are located at both inversion *2z* ^3^ breakpoints. The arrangement of TSDs and the chimeric nature of both *GalileoN* copies provide unequivocal evidence that this transposon generated inversion *2z* ^3^ by ectopic recombination. The *2z* ^3^ proximal breakpoint lies ∼24 kb downstream of the *proboscipedia* gene in a poorly annotated region where two novel genes, *Dlh* and *Mdp*, have been discovered.

## Results

### Physical Mapping of the *2z* ^3^ Inversion Breakpoints in the *D. buzzatii* Genome

**Figure 1:**
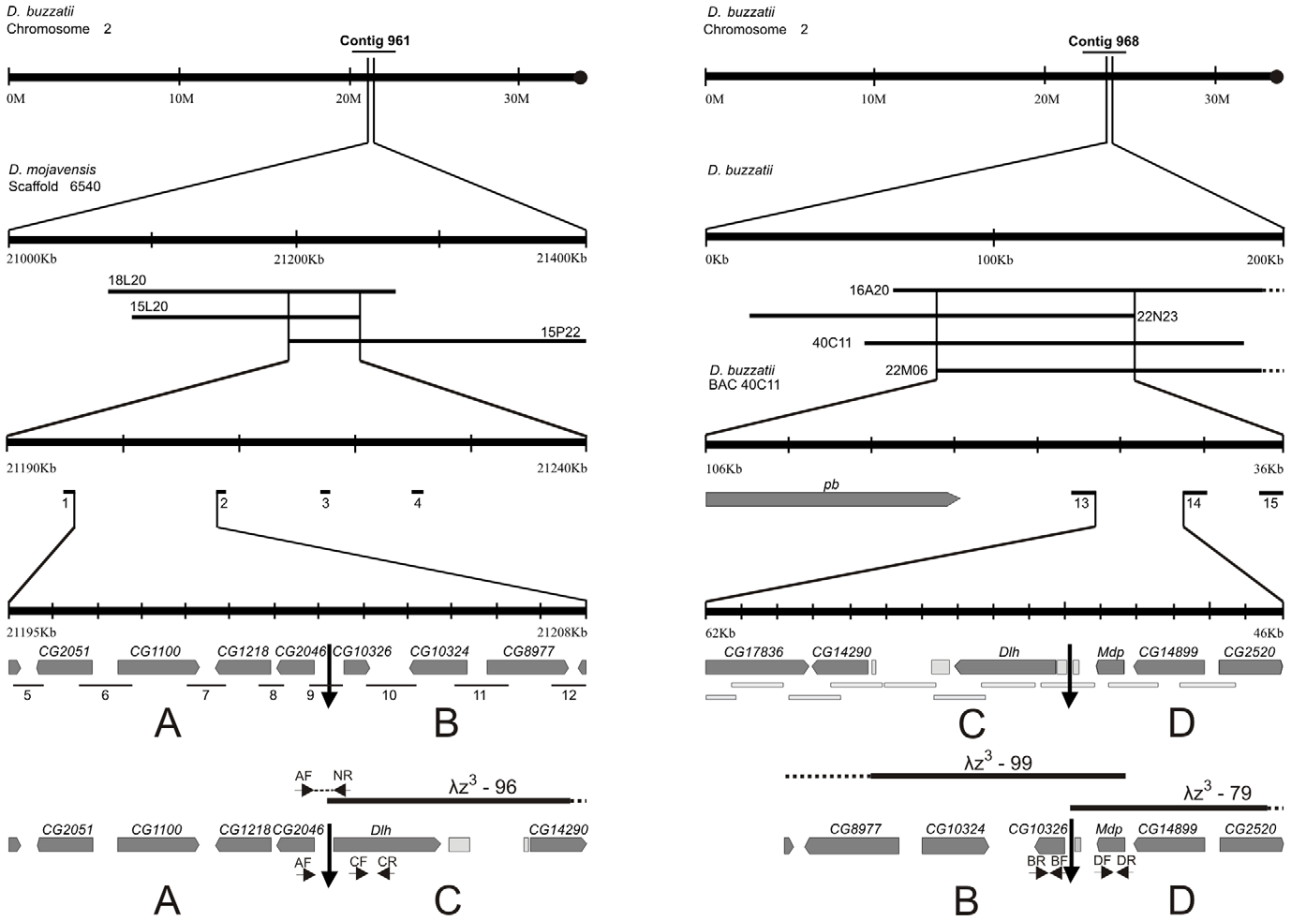
Experimental strategy used for mapping the distal (left) and proximal (right) breakpoints of inversion *2z* ^3^. The segments depicted in each column are ordered from top to bottom in four successive steps with increasing mapping resolution. The distance between consecutive bars stands for 10 Mb, 100 kb, 10 kb and 1 kb, in the four steps, respectively. Line 1: Relative position of the contigs on the physical map of *D. buzzatii* standard chromosome 2. Line 2: Relative position of the BAC clones encompassing the distal breakpoint (left) and the proximal breakpoint (right). Line 3: Position of the PCR probes used to pinpoint the breakpoints within the overlapping segment of BAC clones. Line 4: Genes located in the breakpoint regions of the non-inverted chromosome (designated as AB and CD) are represented by dark grey rectangles with a pointed end indicating the direction of transcription and TEs by light grey rectangles. Short numbered segments under the genes in the distal breakpoint region (left) represent intergenic regions amplified by PCR and grey bars below the genes in the proximal breakpoint column (right) correspond to plasmid subclones of BAC 40C11. Line 5: Genes located in the breakpoint region of the inverted chromosome (designated as AC and BD). Thick lines above the inverted chromosome represent the lambda clones isolated during the cloning of the *2z^3^* breakpoints. Small horizontal arrows represent PCR primers (e.g. AF, NR, …). Vertical arrows mark the location of the breakpoints. Note that there is a reversal of orientation between lines 1 and 2 in the distal breakpoint (left). The reason is inversion *2z* ^3^ took place in a *2j* chromosome and not in the standard chromosome 2 represented in line 1. See Materials and Methods for details.

Previous cytological observations in *D. buzzatii* located the distal and proximal breakpoints of inversion *2z* ^3^ near chromosome 2 bands 2E4c and 2F1c, respectively [63], [68]. We used the BAC-based physical map of the *D. buzzatii* genome [69] and the available genome sequence of the related species *D. mojavensis* [70] to pinpoint the *2z* ^3^ distal breakpoint in the intergenic region between *CG2046* and *CG10326* (see Figure 1 left and Materials and Methods for details). A detailed physical map of the *D. buzzatii* chromosomal region encompassing the *2z* ^3^ proximal breakpoint had been constructed in a previous study [67] and one of the four BAC clones bearing the breakpoint (BAC 40C11) was already fully sequenced and annotated. We mapped the proximal breakpoint within the gene *lodestar* (*lds*) that had been tentatively annotated in that region of BAC 40C11 (see Figure 1 right and Materials and Methods). This annotation was put into question by the subsequent annotation of the *D. mojavensis* genome [70] and a close scrutiny of the region (see below) revealed the presence of two novel genes that we have named *Dlh* and *Mdp*. The *2z* ^3^ proximal breakpoint falls in the intergenic space between them.

### Breakpoint Sequences in the Non-Inverted Chromosomes

**Figure 2:**
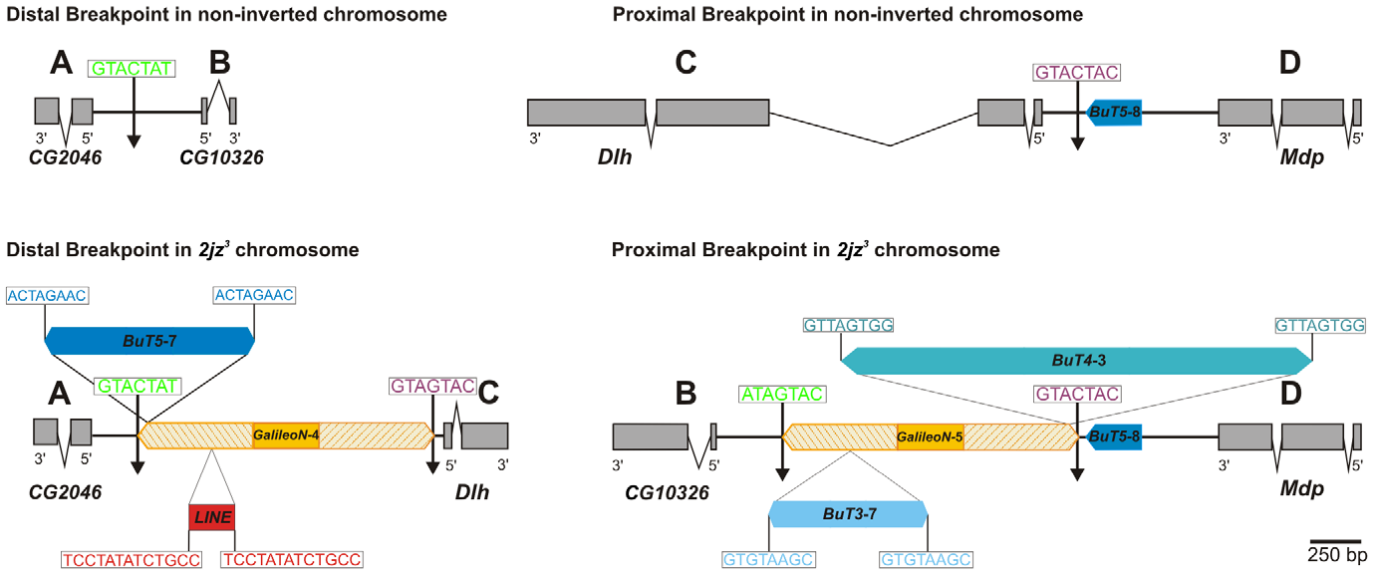
Schematic representation of the structures found at the distal and proximal breakpoint of inversion *2z* ^3^ in *D. buzzatii*. Thick lines represent the single-copy A, B, C and D sequences. Coding regions of genes are represented as grey boxes with an arrow that indicates their orientation. Transposable elements are represented as coloured boxes with pointed ends. The different copies of *GalileoN* have been numbered sequentially following the order of the copies previously described [61]. Vertical arrows indicate the location of the breakpoints. Target site duplications flanking TE insertions are shown in boxes above them.

Following previous sequence analyses of inversion breakpoints [37], [44], the distal and proximal breakpoint regions of *2z* ^3^ were designated as AB and CD in the non-inverted chromosomes (*2st* or *2j*) and as AC and BD in the inverted chromosome (*2jz^3^*). Using primers designed in the *D. mojavensis* genome, we amplified and sequenced 1,022 bp of the distal breakpoint region (AB) between genes *CG2046* and *CG10326* in three *2st* lines and five *2j* lines from diverse geographic origins. In line st-1, the AB sequence comprises 281 bp of gene *CG2046*, 163 bp of gene *CG10326* and the 578-bp intergenic region (Figure 2) including an (AT)_23_ microsatellite (272 bp away from the start codon of *CG2046*). No structural variation was found in the AB region between the eight non-inverted lines except for the number of repeats in the microsatellite (between 16 and 24).

The proximal breakpoint (CD) was localized in the *Dlh* - *Mdp* intergenic region (Figure 2). In line st-1, the intergenic region between these two genes is 1,102-bp long and includes two TE fragments: a 296-bp fragment of *GalileoN* (element *Galileo*, subfamily *Newton*), and a 202-bp fragment of *BuT5* (an unclassified *D. buzzatii* transposon [60]). The CD region was amplified by PCR and sequenced in seven non-inverted lines besides st-1. The CD sequence (1,771 bp) includes 238 bp of gene *Dlh* and 337 bp of gene *Mdp*. All seven lines contained the *BuT5* fragment but only one (j-19) contained the *GalileoN* fragment.

**Figure 3:**
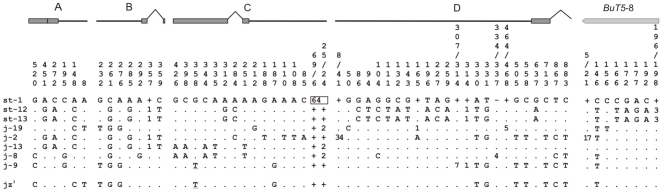
Nucleotide polymorphism at the breakpoints of inversion *2z* ^3^ in inverted and non-inverted chromosomes. For each region, nucleotide positions are numbered taking the breakpoint as start points. The sequence of line st-1 is taken as reference for the A, B, C and D regions, and the *BuT5*-8 insertion. Positions with nucleotides identical to the reference sequence are indicated by a dot. The nucleotide substitution generating a premature stop codon in *Dlh* exon 2 is shown underlined. Insertions and deletions are represented by minus and plus signs in the reference sequence, respectively, and a number in the line with the insertion or deletion indicating its size in nucleotides. In the case of deletions in st-1, a plus sign was added is in the rest of lines, indicating that this sequence is present. Deletions including more than one position of the reference line are included in rectangles. Exons, introns and intergenic regions are not drawn to scale. Variation in the *BuT5*-8 insertion is represented separately from region D.

**Figure 4:**
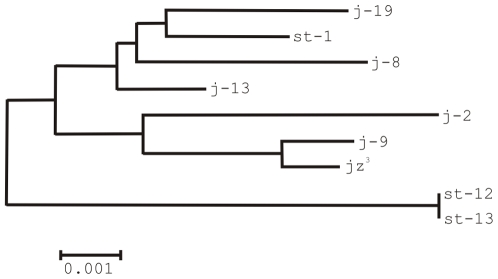
Neighbour-joining phylogenetic tree of the ABCD breakpoint sequences excluding the (AT)_16–24_ microsatellite and the TE insertions.

Levels of nucleotide variation in the *2z* ^3^ breakpoint regions were estimated from the AB and CD sequences of the eight lines without the inversion (Figure 3 and Table S1). Overall, 2,422 bp were analyzed comprising 719 bp of coding sequence, 1,501 bp of non-coding sequence (introns and intergenic segments) and 202 bp of the *BuT5* insertion. Coding and non-coding sequences were analyzed separately. Both the (AT)_16–24_ microsatellite and the polymorphic *GalileoN* insertion were excluded from the analysis. Besides this *GalileoN* insertion, one small insertion of 4 bp and 9 deletions (ranging in size from 1 to 64 nucleotides) were observed in the set of eight lines. Non-coding sequences contain 33 segregating sites (10 in AB and 23 in CD), coding sequences 12 and the *BuT*5 insertion six (Figure 3). Nucleotide diversity [71] values in the different regions are given in Table S1 and a neighbour-joining phylogenetic three built with the non-coding sequences of the single-copy breakpoint regions (ABCD) is shown in Figure 4.

Results can be summarized as follows. First, diversity level does not vary significantly between *2st* and *2j* chromosomes in AB and CD non coding regions (based on the heterogeneity test [72], χ^2^_L_ = 1.69, df = 1, 0.5<*P*<0.1 and χ^2^_L_ = 1.72, df = 1, 0.5<*P*<0.1, respectively). In addition, the lines of the two arrangements appear intermingled in the phylogenetic tree (Figure 4). Second, pooling the eight lines, diversity level of polymorphism of the CD non-coding region is more than two times higher than that of AB non-coding region (π = 0.01391 and π = 0.00567, respectively) and the difference is statistically significant (χ^2^_L_ = 19.23, df = 1, *P*<0.001). This latter result was corroborated by computer simulations. Finally, the level of polymorphism is lower in coding sequences. Three of the sampled genes contain a total of 6 synonymous polymorphisms and 6 amino acid replacement polymorphisms whereas the fourth (*CG10326*) does not present any segregating sites (Figure 3). One of the replacement polymorphisms generates a stop codon in exon 2 of *Dlh* in one of the lines (Figure 3).

### Breakpoint Sequences in the Inverted Chromosome

To isolate the AC and BD regions of the inverted arrangement, two *2jz* ^3^ lambda genomic libraries were screened with appropriate probes from regions C, D and B (see Materials and Methods). Two positive clones were isolated with probe C. *In situ* hybridization of these clones to *2j* chromosomes produced an intense signal at the proximal breakpoint and weak additional signals in multiple sites. This indicates that these clones bear sequences from region C but do not span the complete distal breakpoint region (AC) and also that they contain repetitive sequences. Clone λz^3^-96 was selected for subcloning because its insert reached further away in direction to the breakpoint, and subclones containing the fragments located closest to the breakpoint were sequenced (Figure 1). This provided the sequence of region C and also repetitive sequences inserted at the breakpoint junction but not region A. The rest of the AC region was isolated by PCR using two primers, NR located at the end of the λz^3^-96 clone and AF anchored in gene *CG2046* from region A (Figure 1). The resulting PCR product was sequenced (1,072 bp) and assembled together with the insert of clone λz^3^-96 to complete the sequence of the distal breakpoint AC.

Three positive clones were isolated with probe D. These clones produced an intense signal at the proximal breakpoint when hybridized to *2j* chromosomes but also weak additional signals in multiple locations. This indicates that these clones bear sequences from region D but do not span the complete proximal breakpoint region (BD) and also that they contain repetitive sequences. Clone λz^3^-79 containing the longest insert was subcloned and subclones of interest sequenced confirming that it did not contain sequences from region B (Figure 1). Moreover, this time the remaining part of the sequence could not be amplified by PCR, so we screened the two *2z* ^3^ lambda libraries with a probe from region B. Three additional lambda clones were isolated and tested by PCR for the presence of the genes at each side of the breakpoint. Clone λz^3^-99 was chosen as it contained the genes *CG10326* and *Mdp*, located in regions B and D, respectively. Southern blot analysis revealed that in λz^3^-99 clone these markers were separated by ∼5 kb, therefore it was completely sequenced and the sequence of the proximal breakpoint (BD) was determined.

In total, we sequenced 4,067 bp and 12,520 bp from the distal (AC) and proximal (BD) breakpoint regions in a chromosome with the *2z* ^3^ inversion. Comparison of these sequences with the breakpoint regions in non-inverted chromosomes (AB and CD) allowed us to locate the precise site of the breakpoint junctions within the intergenic regions (Figure 2). This comparison also revealed that there are no fixed nucleotide substitutions between inverted and non-inverted chromosomes (Figure 3). In the phylogenetic tree the *2jz* ^3^ chromosome does not form a separate lineage and appears to be closest to the j-9 line (Figure 4), with which it shares the premature stop codon in *Dlh* exon 2 (Figure 3). Relatively large insertions were found at the AC (2,870 bp) and BD (4,786 bp) junctions that were not present in non-inverted chromosomes (Figure 2). These insertions are composed of several TE insertions, most of them similar to elements previously characterized in *D. buzzatii* [38], [60]. The detailed TE content of the breakpoint insertions is summarized in Table 1.

**Table 1:** Transposable elements found at the breakpoint regions of inversion *2z^3^* in *D. buzzatii*.

Breakpoint Region	Family-copy	Size (bp)	TIR (bp)	TSD (bp)
AC	*GalileoN-*4	1541	575/610	7
AC	*BuT5*-7	1039	3/3	8
AC	*LINE*-like	261	-	13
BD	*GalileoN*-5	1533	606/580	7
BD	*BuT4*-3	2441	23/24	8
BD	*BuT3*-7	795	23/23	8
C	*GalileoN*-6	296	10/10	7
D	*BuT5*-8	202	3	-

The 2,870-bp insertion in the AC junction comprises a copy of *GalileoN* (*GalileoN*-4) with two nested insertions: a copy of *BuT5* (*BuT5*-7) flanked by 8-bp TSDs and a 261-bp copy of a *LINE*-like element (Figure 2). The latter copy has no apparent ORF and no significant sequence homology with described elements. We have classified this insertion as a partial *LINE*-like element because it shows a 41-bp long polyA tail and two flanking 13-bp TSDs. The 4,786-bp insertion in the BD junction comprises also a copy of *GalileoN* (*GalileoN*-5) with two other nested TE insertions (Figure 2): a copy of *BuT4* (*BuT4*-3) flanked by 8-bp TSDs and a copy of *BuT3* (*BuT3*-7) flanked also by 8-bp TSDs. *BuT4* was previously classified as a Class II element of the *hAT* superfamily [60]. This is corroborated by the 87% nucleotide identity observed between this copy of *BuT4* and *Homo7*, a *hAT* element recently described in *D. mojavensis* [73]. This copy of *BuT4* includes a 1774-bp segment with a 87.7% identity to *Homo7* transposase-encoding ORF.

The two *GalileoN* copies inserted at the breakpoint junctions (*GalileoN*-4 and *GalileoN*-5) have relatively long TIRs (Table 1) and are very similar to copies of the subfamily *Newton* previously described in *D. buzzatii* [60]. Upon insertion, *Galileo* generates 7-bp TSDs with the consensus sequence GTAGTAC [61], [62]. The 7-bp sequence flanking *GalileoN*-4 in region C (GTAGTAC) is the reverse and complementary version of the 7-bp sequence flanking *GalileoN*-5 in region D (GTACTAC). Likewise, the 7-bp sequence flanking *GalileoN*-4 in region A (GTACTAT) is the inverted and complementary version of that flanking *GalileoN*-5 in region B (ATAGTAC). Only one single copy of the 7-bp sequence GTACTAT is present at the distal breakpoint (AB) and one copy of the target sequence GTACTAC is found at the proximal breakpoint (CD) in the non-inverted chromosomes. This pattern of exchanged TSDs is consistent with ectopic recombination as the mechanism that generated the *2z* ^3^ inversion (see Discussion).

### Two Novel Drosophila Genes

The proximal breakpoint of inversion *2z* ^3^ was located within BAC 40C11 in the genomic region between genes *CG14899* and *CG14290*. This *D. buzzatii* chromosome 2 region had been tentatively annotated as containing a single five-exon gene orthologous to *D. melanogaster lds* [67]. However, only three of the five exons of the *D. buzzatii* gene model showed significant homology with *Dmel\lds*. We failed to corroborate the structure of the putative *D. buzzatii lds* gene by RT-PCR using primers anchored in exons 1 and 5. In addition, the sequencing and annotation of 12 Drosophila genomes [70] revealed that in *D. mojavensis*, the closest species to *D. buzzatii*, the *lds* ortholog is located in a distant chromosome 2 region casting doubts on the *D. buzzatii* annotation. These observations prompted a detailed comparative analysis of the 7.5-kb *D. buzzatii* region between genes *CG14899* and *CG14290* with the homologous regions in *D. mojavensis* and *D. virilis* and a search for RNA expression by RT-PCR (see Materials and Methods).

The results lead us to discard the *lds* annotation and discover two novel Drosophila genes, whose main characteristics are described in Table S2. In *D. buzzatii*, the gene that we have named *MADF domain protein* (*Mdp*) is composed of three exons and two introns with a total length of 794 bp (Table S2). The coding sequence is 651-bp long and encodes a 216-aa protein with a MADF domain (Figure S2). *Mdp* has been found also in *D. mojavensis* and *D. virilis* with a similar structure, although a somewhat longer coding sequence in *D. virilis* and a stop codon in position 142 of the third exon in *D. mojavensis*. As expected from the phylogenetic relationships, nucleotide identity and amino acid identity were higher with *D. mojavensis* (82.5% and 76.3%, respectively) than with *D. virilis* (70.4% and 60.9%, respectively). The overall codon-based Z-test of purifying selection shows highly significant results (Z = −10.15, P<10^−6^) and the ratio of synonymous to non-synonymous substitutions (Ka/Ks  = 0.22) shows a moderate degree of functional constraint. The second gene has been named *DEAD-like helicase* (*Dlh*) and in *D. buzzatii* it comprises four exons and three introns with a total length of 2,826 bp. The coding sequence is 1,554-bp long and encodes a 517-aa protein with a SNF2-related or DEAD-like helicase N-terminal domain and a DNA/RNA helicase C-terminal domain (Figure S3). This gene is also present in *D. mojavensis* with a similar structure, but could not be found in *D. virilis* (Table S2). Nucleotide identity of the coding sequence (76.8%) and amino acid identity of the protein (64.5%) support orthology. The estimated ratio Ka/Ks was relatively high (0.48), but significantly lower than 1 (Z = −5.56, P = 2×10^−7^) suggesting that this is a relatively fast evolving gene.

## Discussion

### Inversion *2z* ^3^ Was Generated by Ectopic Recombination between *Galileo* Copies

Many studies have shown the potential of TEs to induce chromosomal rearrangements in experimental Drosophila populations implicating retrotransposons (e.g. *BEL*, *roo*, *Doc*, and *I*) as well as transposons (e.g. *P*, *hobo*, and *FB*) [74]. In contrast, the evidence for the involvement of TEs in the generation of natural Drosophila inversions, i.e. those effectively contributing to adaptation and/or evolution of natural populations, is scarce (see Introduction). We have previously found that the cut-and-paste transposon *Galileo* was involved in the generation of two polymorphic inversions of *D. buzzatii*, *2j* and *2q* ^7^ [37], [38]. Here we have isolated and sequenced the breakpoints of another polymorphic inversion of *D. buzzatii*, *2z* ^3^. Our results provide the most compelling evidence for the participation of *Galileo* in the generation of Drosophila inversions and for ectopic recombination as the responsible mechanism.

Several TE insertions were found at the breakpoint regions in the chromosome with the *2z* ^3^ inversion that were not present in non-inverted chromosomes (Table 1). Remarkably, only *GalileoN* was present at the two breakpoint junctions. This fact and the evidence presented below indicate that *GalileoN* is the element responsible for the generation of the *2z* ^3^ inversion. Two other TE insertions, *BuT5* and *LINE*-like, were found nested within the *GalileoN* copy in the distal breakpoint and another two, *BuT3* and *BuT4*, within the *GalileoN* copy in the proximal breakpoint. These four TE insertions are present at a single breakpoint junction only and each of them is flanked by identical direct TSDs. Thus, they are unlikely to be responsible for the generation of the inversion and are best interpreted as secondary colonizers of the breakpoint regions (see below). Another two TE fragments (*BuT5* and *GalileoN*) are present in the proximal breakpoint region (but not in the junction) of non-inverted chromosomes and thus can not be involved in the generation of the inversion either.

Two processes can explain the induction of chromosomal inversions by TEs: ectopic recombination [12], [74] and aberrant transposition [13]–[17]. Ectopic recombination requires the presence in the parental chromosome of two homologous TE copies inserted in opposite orientation at different sites. After the inversion is generated, two chimeric TE copies are expected to be found flanking the inverted segment with their TSDs exchanged. On the other hand, two transposon copies may participate in an aberrant transposition event, by which a hybrid element formed by the 5′ end of one copy and the 3′ end of the other copy transposes to a new chromosomal site. The outcome of this process is an inversion flanked by two transposon copies in opposite orientation accompanied by deletions or duplications when the original copies were inserted at separate chromosomal sites (Figure S1). The lack of any deletions or duplications and the pattern of TSDs in the *2z* ^3^ breakpoints allow us to reject this latter possibility. However, we must consider the possibility of an aberrant transposition with the two original transposon copies located at the same chromosomal site (hybrid insertion model). The outcome in this case (Figure S1 A) is strikingly similar to that of ectopic recombination except for the fact that the two TE copies flanking the inversion are identical under the hybrid element insertion model but chimeric under the ectopic recombination [38].

**Figure 5:**
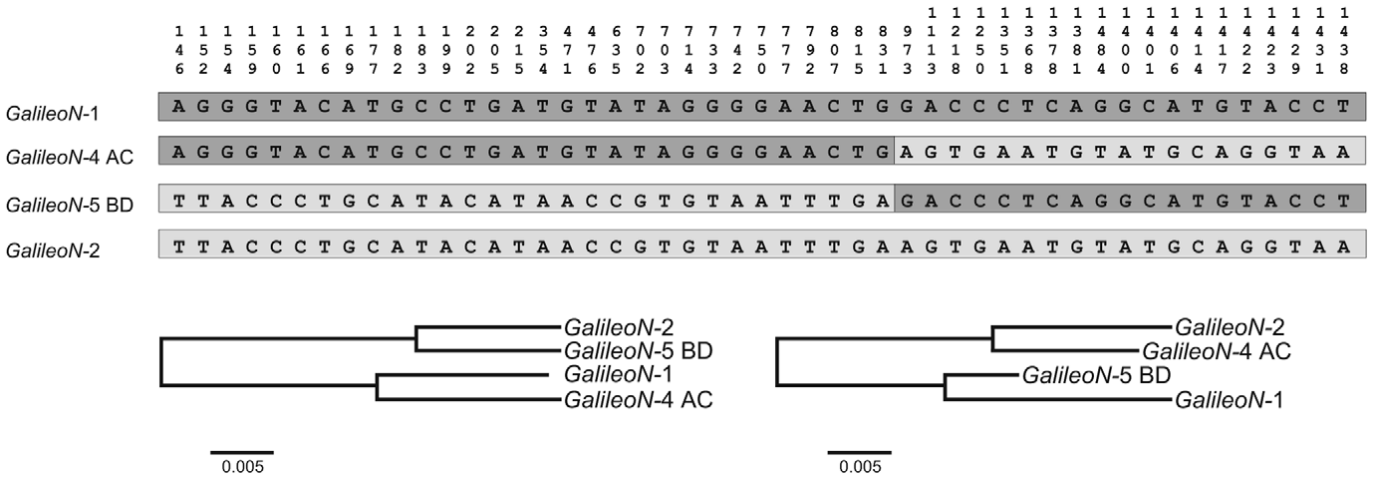
Chimeric structure of the two *GalileoN* copies (*GalileoN*-4 and *GalileoN*-5) observed at the breakpoints of inversion *2z* ^3^. *GalileoN*-1 and *GalileoN*-2 were found in a previous study [60]. Top: Nucleotides present in the four *GalileoN* copies at 53 variable sites are shown. Bottom: Neighbor-joining phylogenetic trees of the *GalileoN* sequences built separately for the two portions of the sequence: sites 1–824 (left) and sites 966–1567 (right).

The two *GalileoN* copies found in the *2z* ^3^ breakpoints (named *GalileoN*-4 and *GalileoN*-5) have similar sizes and structures, with relatively long TIRs and a middle segment oriented in opposite direction in the two copies, and show a high similarity with two other copies previously described (*GalileoN*-1 and *GalileoN*-2) [60], [61]. Each of the latter two copies was flanked by perfect 7-bp TSDs generated upon insertion. By contrast, the 7-bp duplications flanking the *GalileoN* copies at the *2z* ^3^ breakpoints are exchanged (Figure 2 and Results). In the non-inverted chromosomes, only one copy of the corresponding 7-bp target sequence is detected at each breakpoint (Figure 2). These observations are consistent with the presence of two *GalileoN* insertions in the parental chromosome and the generation of the *2z* ^3^ inversion by ectopic recombination between them, but does not rule out the hybrid element insertion model (see above). Further evidence was revealed by comparing the nucleotide sequence of the TIRs within and between *GalileoN* copies. *GalileoN*-1 and *GalileoN*-2 possess TIRs >99% identical within each copy but ∼7% divergent between copies (Table 2). In contrast, *GalileoN*-4 and *GalileoN*-5 show TIRs that are ∼6% divergent within each copy but >99% identical between copies (Table 2). These results suggest that both *GalileoN*-4 and *GalileoN*-5 are chimeric. A closer scrutiny of the four *GalileoN* copies revealed a striking pattern and led to the same conclusion (Figure 5). In 33 variable sites, from position 1 through 824, the nucleotide present in *GalileoN*-4 is identical to that in *GalileoN*-1 and the nucleotide present in *GalileoN*-5 is identical to that in *GalileoN*-2 (Figure 5 top). The situation is completely reversed for 20 variable sites from position 966 to the end of the element where the nucleotide present in *GalileoN*-4 is identical to that in *GalileoN*-2 while that in *GalileoN*-5 is identical to that in *GalileoN*-1 (Figure 5 top). Phylogenetic analyses of the four sequences carried out separately for the two portions of the element (Figure 5 bottom) and the maximum chi-square method (χ^2^ = 53.00, df = 1, P<1×10^−7^) [75], [76] corroborated the chimeric structure of *GalileoN*-4 and *GalileoN*-5. These observations provide strong support for the ectopic recombination model and suggest that the recombination event that gave rise to the *2z* ^3^ inversion took place within 141-bp of the middle segment between positions 825 and 965 of *GalileoN* (Figure 5). The absence of *GalileoN* insertions in the analyzed non-inverted chromosomes should be no surprise because insertions of actively transposing families are expected to be present at low population frequencies under transposition-selection balance [77], [78] and we sampled just a few non-inverted chromosomes.

**Table 2:** Nucleotide divergence between the TIRs of four *GalileoN* copies.

TIR	1R	2L	2R	4L	4R	5L	5R
1L	**0.0018**	0.0670	0.0670	0.0326	0.0708	0.0552	0.0727
1R		0.0690	0.0690	0.0345	0.0728	0.0271	0.0747
2L			**0.0071**	0.0234	0.0591	0.0514	0.0234
2R				0.0591	0.0234	0.0514	0.0234
4L					**0.0628**	0.0071	0.0647
4R						0.0552	0.0036
5L							**0.0570**

TIR number indicates the *GalileoN* copy, and L and R correspond to 5′ TIR and 3′TIR, respectively; values in boldface correspond to the comparisons between TIRs of the same copy.

We can conclude that the three polymorphic inversions of *D. buzzatii* studied so far, *2j*, *2q* ^7^ and *2z* ^3^, have been generated by the same TE family, *Galileo*, and very likely by the same molecular mechanism, ectopic recombination. In all three cases, after the generation of the inversion, many TE copies have accumulated at the breakpoint regions, which became hotspots for secondary TE insertions (Table 3). This accumulation is probably a consequence of the reduction of recombination in these regions [79], [80] that protects TE copies from being eliminated by deleterious ectopic exchanges [77], [78]. It is intriguing though that the 40 TE copies associated with inversion breakpoints in *D. buzzatii* belong to a limited set of nine TE families (Table 3). All of them but one (the *LINE*-like element in the distal breakpoint of inversion *2z* ^3^) are Class II elements: *ISBu* elements are Helitrons [81] and the remaining elements are cut-and-paste transposons [82]. This enrichment of breakpoint regions in specific TE families may be due (1) to the fact that these TE families were among the most transpositionally active elements in the *D. buzzatii* genome when the opportunity window for insertion was open, and/or (2) to insertional preference [83].

**Table 3:** Number of TE copies found in the breakpoint regions of three D. *buzzatii* polymorphic inversions.

Inversion	*2j*		*2q* ^7^		*2z* ^3^		TOTAL
Breakpoint region a	D	P	D	P	D	P	D+P
*Galileo*	4	5	1	3	1	1	16
*BuT1*		1					1
*BuT2*		1					1
*BuT3*	2	3		1		1	7
*BuT4*	1					1	2
*BuT5*		1	1	4	1	1	8
*BuT6*			1				1
*ISBu*	3	1					4
*LINE*-like					1		1
TOTAL	10	12	3	8	3	4	40

Number of chromosomal lines investigated: 30 for *2j*, 6 for *2q* ^7^ and 1 for *2z* ^3^. Data from [38], [60] and this work.

a D  =  distal; P  =  proximal.

Because many different TE families are able to induce chromosomal rearrangements in Drosophila [74], the question arises as to why the three polymorphic *D. buzzatii* inversions should be generated by the same TE family, namely *Galileo*. The frequency of ectopic recombination should increase with copy number and length, and this prediction is borne out by the data ([78], D. Petrov, personal communication). In the *D. melanogaster* genome, at least 121 TE families are present [84], [85]. A total of 996 copies from 81 families were annotated in the euchromatin of the sequenced genome (excluding the proximal 2 Mb where TEs regularly accumulate) and copy number per family varied between 1 and 124 with an average of 12.3 [84]. Although no detailed inventory of the TE families in the *D. buzzatii* genome is yet available, there is no ground for assuming a smaller number of families than in *D. melanogaster*. *Galileo* copy number per genome was estimated as 11.7 in the euchromatic distal-central region of chromosomes (i.e. excluding the dot and pericentromeric regions) [61]. The analogous figure for *BuT5* is 11.4 copies per genome and lower values were estimated for another five *D. buzzatii* transposons [83]. In summary, *Galileo* copy number does not seem particularly high in the *D. buzzatii* genome, although more data is needed. Length of *Galileo* copies is not unusual either. The canonical copy is ∼5.4 kb long [62] but most copies are non-autonomous and much shorter. Average length (± SD) of a combined sample of 23 non-autonomous copies of *GalileoG*, *GalileoN* and *GalileoK* is 953 bp (±640 bp) [61]. In *D. melanogaster*, the average length of the TE copies annotated by [84] was 2.9 kb.

Two characteristics of *Galileo* can explain its primary role in the generation of rearrangements by ectopic recombination: (1) its transpositional activity; and (2) its unusual structure. *Galileo* belongs to the *P* superfamily of TIR transposons and is likely to transpose by a cut-and-paste mechanism similar to that of the *D. melanogaster P* element [86], [87]. This transposition mechanism involves the binding of the transposase to the element TIRs and the excision of the element generating a DSB at the donor site followed by the integration of the element into a different chromosomal site. Hence DSBs produced during normal or aberrant transposition events may provide the initial step for ectopic recombination events. The accumulation of *Galileo* copies after the generation of inversions *2j* and *2q* ^7^ (Table 3) indicates that *Galileo* is (or has been recently) active in the genome of *D. buzzatii*. Nevertheless, unless *Galileo* has an unusually high transposition rate, this explanation is insufficient because *Galileo* is not the only TE family transpositionally active in the *D. buzzatii* genome (at least another eight TE families must be active; Table 3).

The participation of *Galileo* in the generation of inversions may be also related to its unusual structure with up to 1.2-kb long TIRs [61], [62]. The two *GalileoN* copies involved in the generation of the *2z* ^3^ inversion have ∼575 bp long TIRs separated by a ∼350 bp long middle segment (Table 1). This kind of spaced inverted repeat sequences is well known to form stem-loop structures in single-stranded DNA or cruciform structures in double-stranded DNA and induce DSBs and rearrangements in a wide variety of organisms [88]-[93]. Generation of DSBs by these secondary structures may be due to the fact that they are substrates for nuclease cleavage or because they interrupt replication fork progression [94], [95]. In *D. melanogaster*, *Foldback* (FB) elements, which also present very long TIRs and induce secondary structures, are known to cause rearrangements at a high rate in the laboratory [96], [97]. We propose that the long TIRs of *Galileo* induce the formation of secondary structures and DSBs at high rate and this contributes to its unique capacity to generate chromosomal inversions. The fact that the recombination event that generated inversion *2z* ^3^ took place in the middle segment of *GalileoN* seems consistent with nuclease cleavage at the loop.

### Functional Consequences of the *2z* ^3^ Inversion

Inversion *2z* ^3^ seems to have a recent origin as no fixed nucleotide substitution was observed in the breakpoint regions between non-inverted and inverted chromosomes (Figure 3). This is in clear contrast with the ∼1 Myr and ∼0.5 Myr old inversions *2j* and *2q* ^7^ where 17 and 14 fixed nucleotide substitutions were observed, respectively [38], [60]. The monomorphism of the *α-esterase5* gene in *2jz* ^3^ chromosomes is also consistent with a recent origin of inversion *2z* ^3^ [98]. In spite of being a very young inversion, *2z* ^3^ exhibits a widespread distribution in natural populations (see Introduction), suggesting that it must have a considerable selective value. In Argentina, the frequency of *2jz* ^3^ is significantly correlated with latitude, a putatively selective pattern [65]. Furthermore, selection component analyses and biometrical studies they all have detected significant effects of *2jz* ^3^ chromosomes [99]–[102]. One possible explanation for its adaptive advantage is provided by the position effect hypothesis, which proposes that the localization of the inversion breakpoints near or inside genes could affect their function or expression profile by disrupting their coding regions or causing changes in the promoter and regulatory regions [103], [104]. Another factor that could affect the expression of genes adjacent to the breakpoints is the presence of TEs in these regions as they have been shown to alter gene expression in different ways [103], [105], [106].

The *2z* ^3^ proximal breakpoint lies in a region previously sequenced where a gene named *lodestar* (*lds*) had been tentatively annotated [67]. A comparative analysis with other Drosophila genomes and expression experiments by RT-PCR discarded the *lds* annotation and has unveiled two novel genes flanking the inversion breakpoint, *Dlh* in region C and *Mdp* in region D. Three observations suggest that these two genes are fully functional. (*i*) In *D. buzzatii*, both genes are expressed throughout the whole life cycle, although they present slightly different expression patterns (results not shown). (*ii*) Their overall structure and encoded protein sequence are conserved in at least another Drosophila species (Table S2). (*iii*) Both genes are evolving under purifying selection with Ka/Ks ratios significantly different from 1 (strict neutrality). The relatively short intergenic region (796 bp) and the close proximity of the proximal breakpoint to the initiation codon of *Dlh* (118 bp) suggest that the inversion might be affecting the expression of *Dlh* and/or *Mdp*, a question that deserves further work.

In *D. buzzatii*, the Hox gene complex is split in three portions: *proboscipedia* (*pb*)-*Ultrabithorax* (*Ubx*), *abdominalA* (*abdA*)*-AbdominalB* (*AbdB*) and *labial* (*lab*) [66], [67]. We analyzed the breakpoints of inversion *2z* ^3^ in part because of the cytological vicinity of the *2z* ^3^ proximal breakpoint to the *pb-Ubx* portion of the Hox gene complex [63], [66], [67]. Our results show that the *2z* ^3^ proximal breakpoint lies outside of the Hox gene complex ∼23.7-kb downstream of *pb*. The segment that separates the *2z* ^3^ proximal breakpoint from *pb* contains three genes, *CG17836, CG14290* and *Dlh*. It seems unlikely that the *2z* ^3^ proximal breakpoint altered the regulatory sequences or the expression pattern of *pb* because the *lab-pb* split that took place much nearer the 3′ end of *pb* in the ancestor of the repleta group did not [67]. Nevertheless, the *pb-Ubx* portion of the Hox gene complex is located within the inverted segment and thus the *2z* ^3^ inversion relocates these genes to a much more distal region within chromosome 2. Whether this change in the chromatin environment has had any effect on the expression of Hox genes remains an open question.

## Materials and Methods

### Drosophila Stocks

Nine lines of *D. buzzatii* homokaryotypic for one of three different chromosome 2 arrangements (*2st*, *2j* and *2jz* ^3^) were used. These lines were isolated from natural populations with different geographical origin: st-1, Carboneras (Spain); st-12, Trinkey (Australia); st-13, Mazán (Argentina); j-2, Carboneras (Spain); j-8, San Luis (Argentina); j-9, Quilmes (Argentina); j-13, Guaritas (Brazil); j-19, Ticucho (Argentina); and jz^3^-2, Carboneras (Spain). The stock of *D. mojavensis* (15081–1352.22, UC San Diego Drosophila Species Stock Center) comes from Santa Catalina Island (California) and is the stock used to sequence the *D. mojavensis* genome [70].

### Probes and *In Situ* Hybridization

DNA from BAC and plasmid clones was extracted by alkaline lysis following standard protocols and used as probes for *in situ* hybridization. All remaining probes were produced by polymerase chain reaction (PCR) amplification of *D. buzzatii* or *D. mojavensis* genomic DNA with different primer pairs. Probes were labelled with biotin-16-dUTP (Roche) by random priming and hybridization to the larval salivary gland polytene chromosomes was carried out according to the procedure described [107]. Intraspecific *in situ* hybridizations with *D. buzzatii* lines and probes were carried out at 37°C while interspecific hybridizations of *D. mojavensis* probes to *D. buzzatii* polytene chromosomes were carried out at 25°C. Hybridization results were recorded as digital images captured with phase contrast Nikon Optiphot-2 microscope at 600× magnification and a Nikon Coolpix 4500 camera. Cytological localization of the hybridization signal was determined using the cytological maps of *D. buzzatii* [63], [69].

### Physical Mapping of the Inversion Breakpoints

We searched the BAC-based physical map of the *D. buzzatii* genome [69] for clones located near the cytological breakpoints and selected eight clones from contig 961 mapping near the distal breakpoint, and seven clones from contig 968 mapping near the proximal breakpoint (Table S3). The fifteen BAC clones were hybridized to the salivary gland chromosomes of one line with the inversion (jz^3^-2) and one line without the inversion (j-9) to identify those clones containing a breakpoint (that should produce two hybridization signals in the first case and a single hybridization signal in the second). Three BAC clones from contig 961 (18L15, 15P22 and 15L20) were found to include the distal breakpoint (Figure S4A), and four clones from contig 968 (22N23, 22M06, 16A20, and 40C11) were found to contain the proximal breakpoint (Figure S4E).

Both ends of each BAC clone bearing the distal breakpoint were sequenced and the sequences mapped onto the genome sequence of *D. mojavensis* using BLASTN (Figure 1 left). The distal breakpoint was located in the overlapping region between the three *D. buzzatii* BAC clones, a segment ∼50-kb long of *D. mojavensis* scaffold_6540 that corresponds to chromosome 2 [108]. To narrow down the position of the breakpoint we chose four genes within this segment (*CG1193*, *CG14906*, *Adk3* and *CG4674*) and used them as probes for *in situ* hybridization to *2jz^3^* chromosomes (Table S4). The *CG1193* probe (marker 1 in Figure 1 left) mapped at the distal breakpoint, outside the inversion, while the other three probes (markers 2, 3 and 4 in Figure 1) hybridized at the proximal breakpoint, indicating that they are located inside the inverted segment. As a result, we located the distal breakpoint in the 13-kb segment between genes *CG1193* and *CG14906* (markers 1 and 2 in Figure 1). Seven genes had been annotated in this segment of *D. mojavensis* chromosome 2 and we designed primers to amplify the intergenic region between each pair of genes in this species, as well as in *D. buzzatii* strains with and without inversion *2z* ^3^. Our rationale was that the intergenic region containing the distal breakpoint would amplify in *D. mojavensis* and in the line with the non-inverted chromosome, but not in the line carrying the inversion. In fact, all the intergenic segments were amplified in the three lines, except that between *CG2046* and *CG10326* (segment 9 in Figure 1 left) which failed to amplify in the line carrying the inversion. To corroborate this observation, PCR products amplified using the primers 8F-8R, 9F-9R and 10F-10R were used as *in situ* hybridization probes to chromosomes with the inversion, and they produced the expected results (Figure S4B, C and D). Therefore, the distal breakpoint of inversion *2z* ^3^ was located in the ∼600-bp region between genes *CG2046* and *CG10326* of *D. mojavensis*.

One of the four BAC clones bearing the *2z* ^3^ proximal breakpoint (BAC 40C11) was already fully sequenced and annotated and a physical map of the region was built using sequence tagged sites (STSs) [67]. This map allowed us to locate the proximal breakpoint in the ∼70-kb region of overlap between the four clones (Figure 1 right). Three STS markers generated in this region were amplified and hybridized to *2jz^3^* chromosomes, in order to further delimit the region which contains the proximal breakpoint (Figure 2 right). One marker (number 13 in Figure 1 right) hybridized to the distal breakpoint and therefore was located inside the inversion, whereas the other two (markers 14 and 15 in Figure 2 right) mapped on the region of the proximal breakpoint, indicating that they are located outside the inverted segment. As a result, the proximal breakpoint could be narrowed down to a 16-kb segment between genes *CG17836* and *CG2520* (markers 13 and 14 in Figure 1 right). Ten plasmid subclones from BAC 40C11 which cover this segment were also used for hybridization to inverted chromosomes (Figure S4F, G and H and Table S4), allowing us to locate the proximal breakpoint more precisely in the ∼0.8-kb intergenic region between genes *Dlh* and *Mdp* (Figure 1 right).

### Southern Blot and Screening of Genomic Libraries

Southern hybridization and library screenings were carried out by standard methods [109]. Three different probes amplified from *D. buzzatii* DNA: DF-DR (800 bp), CF-CR (337 bp) and BF-BR (505 bp) were used (Table S5). Probes were labelled by random priming with digoxigenin-11-dUTP under the conditions specified by the supplier (Roche). Hybridization was carried out overnight at 42°C in a standard hybridization solution (Roche). Stringency washes were performed with 0.5x SSC 0.1% SDS solution at 65°C. Two lambda genomic libraries were screened. One library was constructed with DNA derived from *D. buzzatii* line jz^3^-2 using the LambdaGEM-11 vector following manufacturer's instructions (Promega). The second lambda library was derived previously from *D. buzzatii* line jz^3^-4 [60] and was amplified using standard methods [109]. Two positive clones (λz^3^-91 and λz^3^-96) were recovered from the first library with probe CF-CR and six positive clones were recovered from the second library, three with probe DF-DR (λz^3^-77, λz^3^-79 and λz^3^-98) and three with probe BF-BR (λz^3^-99, λz^3^-102 and λz^3^-104). The span of each clone was determined through a combination of PCR, restriction mapping and Southern blotting. DNA fragments of interest from positive phages were subcloned into pBluescript II SK vector (Stratagene).

### PCR Amplification

Polymerase chain reaction was carried out in a volume of 25 µl, including 50–100 ng of genomic DNA, 10 pmol of each primer, 100 µM dNTPs, 1x buffer and 1–1.5 units of Taq DNA polymerase. Temperature cycling conditions were 30 rounds of 30 s at 94°C; 30 s at the annealing temperature, and 30–60 s at 72°C, with annealing temperatures varying from 55 to 60°C depending on the primer pair. Sequences of oligonucleotide primers are given in Table S5.

### RNA Extraction and RT-PCR Amplification

Total RNA was isolated from embryos, larvae, pupae, and adults of the *D. buzzatii* st-1 line using TRIzol (Invitrogen). Total RNA was treated with 1 unit of DNase I (Ambion) for 30 min at 37°C to eliminate DNA contamination. cDNA was synthesized from 1 µg of DNase I-treated RNA by using an oligo(dT) primer (Transcriptor First Strand cDNA Synthesis kit for RT-PCR, Roche). PCR reactions were performed as describe above. To differentiate the size of amplification products, both cDNA and st-1 genomic DNA were used as templates. RT-PCR products were sequenced and their sequences compared with those of genomic DNA to determine exon-intron boundaries (Figures S2 and S3).

### DNA Sequencing and Sequence Analysis

Sequencing was performed in the Servei de Genòmica of the Universitat Autònoma de Barcelona, Macrogen Inc. (Seoul, Korea) and GATC Biotech (Konstanz, Germany). Fragments cloned into pBluescript II SK were sequenced with the M13 universal and reverse primers. PCR products were gel purified using QIAquick Gel Extraction Kit (Qiagen), and sequenced directly with the same primers used for amplification.

Sequences from different lines were aligned with MUSCLE 3.2 [110] and similarity searches in the GenBank/EMBL, Assembly/Alignment/Annotation of 12 related Drosophila species (http://rana.lbl.gov/drososphila/) and FlyBase databases were carried out using BLASTN [111]. Nucleotide variability was estimated by means of the number of segregating sites (S), and the nucleotide diversity (π, average number of pairwise differences per site) using DnaSP (version 4.50.3) software [112]. This software was also used to test for differences in nucleotide variability by means of computer simulations based on the coalescent process. Simulations were carried out given the number of segregating sites and analysing the nucleotide diversity (π) on the genealogy, fixing the options of no recombination to AB region and free recombination to CD region, because AB region mapped inside the *2j* inversion. Interspecific nucleotide and amino acid similarities were estimated with MEGA 4 [113]. The ratios of non-synonymous to synonymous nucleotide substitutions (Ka/Ks) were estimated using Nei-Gojobori method and Jukes-Cantor distance. The null hypothesis that Ka/Ks  =  1 was tested by means of the Z-test of selection. Phylogenetic analyses were also conducted using MEGA 4.

Sequence data from this article have been deposited in the GenBank/EMBL Database Libraries under accession nos. GU132438-GU132454.

## Supporting Information

Figure S1 Chromosomal inversions may be generated by transposons when two ends that are not part of the same transposon participate in an aberrant transposition event to a new site [13]–[17]. Target site duplications (TSD) are indicated by ○ or □ (cooperating TE copies) and Δ (new insertion site). (A) The two TE copies are located at the same site of sister chromatids or homologous chromosomes and share the same TSD (○). The result of the aberrant transposition is an inversion (segment BC) flanked by two TE copies. (B) The two TE copies are inserted at separate sites in the two homologous chromosomes and each has its own TSD (indicated by ○ and □). The aberrant transposition event produces an inversion (segment BC) and a deletion (segment D). (C) The two TE copies are arranged as in (B) but two different element ends are involved. The resulting chromosome carries an inversion (segment BC) and a duplication (segment D). (D) The two TE copies are inserted at separate sites on the same chromatid and each has its own TSD (indicated by ○ and □). The resulting chromosome has an inversion (segment BC) and a deletion (segment D).
(0.02 MB PDF)

Figure S2 Alignment of gene *Mdp* sequences in three Drosophila species. The aligned sequences are: positions 50294–51354 from *D. buzzatii* BAC clone 40C11 (accession number AY900632), positions 6137692–6136590 from *D. mojavensis* scaffold_6540 and positions 5807143–5806092 from *D. virilis* scaffold_12855. Yellow boxes indicate exons with the initial methionine and the final stop codon colored in orange and red, respectively. The premature stop codon found in the *D. mojavensis* sequence is also shown as a red box. Note that there are some parts of the sequence upstream of the coding region that are conserved in the different species suggesting that they may be part of the 5′ UTR or the regulatory regions of the gene. A putative polyA signal determined only on the basis of sequence conservation in the different species is included in a purple rectangle. The blue bar below the alignment indicates the 763-bp fragment amplified by RT-PCR and sequenced in *D. buzzatii* with primer pair DF-DR. The protein sequence encoded by the *D. buzzatii* gene is shown above the alignment. The residues enclosed in a green box correspond to the *MADF* domain found using InterProScan (http://www.ebi.ac.uk/Tools/InterProScan/).
(0.01 MB PDF)

Figure S3 Alignment of gene *Dlh* sequences in two Drosophila species. The aligned sequences are: positions 52175–55219 from *D. buzzatii* BAC clone 40C11 (accession number AY900632) and positions 6136143–6133352 from *D. mojavensis* scaffold_6540. This gene could not be found in the *D. virilis* genome sequence. Yellow boxes indicate exons with the initial methionine and the final stop codon colored in orange and red, respectively. Enclosed in a purple rectangle is the codon in the second exon of the gene that becomes a polymorphic premature stop codon in lines j-9 and jz3-1 by changing from TCA to TAA. No further upstream non-coding sequence could be included in the alignment because of the presence of a polymorphic *GalileoN* insertion in the st-1 line, from which the *D. buzzatii* BAC clone is derived. Bars below the alignment in different shades of blue indicate the three overlapping fragments amplified by RT-PCR and sequenced in D. buzzatii with primer pairs CF-CR (278 bp), CF-RT1R (609 bp) and RT2F-RT2R (1,011 bp). The protein sequence encoded by the *D. buzzatii* gene is shown above the alignment. The residues enclosed in a dark green box correspond to a SNF2-related or a DEAD-like helicase N-terminal domain and the aminoacids in a light green box correspond to a DNA/RNA helicase C-terminal domain. The protein domains have been analyzed using InterProScan ((http://www.ebi.ac.uk/Tools/InterProScan/).
(0.02 MB PDF)

Figure S4 *In situ* hybridization to *D. buzzatii* chromosomes carrying inversion *2z* ^3^ of BAC clones, plasmid clones and PCR probes coming from the distal breakpoint (A-D) and the proximal breakpoint (E-H). A: BAC clone 18L15; B: PCR fragment 10F-10R; C: PCR fragment 9F-9R; D: PCR fragment 8F-8R. E: BAC clone 40C11; F: plasmid clone 9F01; G: plasmid clone 8H04; H: plasmid clone 8D03. Arrows indicate hybridization signals.
(4.84 MB TIF)

Table S1 Nucleotide variability in non-inverted chromosomes. N  =  number of chromosomal lines; m =  number of compared nucleotides.
(0.05 MB PDF)

Table S2 Structure and similarities of two novel Drosophila genes: *MADF domain protein* (*Mdp*) and *DEAD-like helicase* (*Dlh*). NT  =  nucleotide; AA  =  amino acid.
(0.01 MB PDF)

Table S3 BAC clones used for in situ hybridization.
(0.01 MB PDF)

Table S4 Plasmid clones used as probes for *in situ* hybridization to map the proximal breakpoint of the *2z* ^3^ inversion.
(0.01 MB PDF)

Table S5 Sequence of oligonucleotide primers used for PCR amplification.
(0.05 MB PDF)
