# Supplementary figures and images for: The Transposon Galileo Generates Natural Chromosomal Inversions in Drosophila by Ectopic Recombination
Source: PLoS One. 2009 Nov 18;4(11):e7883. doi: 10.1371/journal.pone.0007883 (PMC2775673; doi:10.1371/journal.pone.0007883)

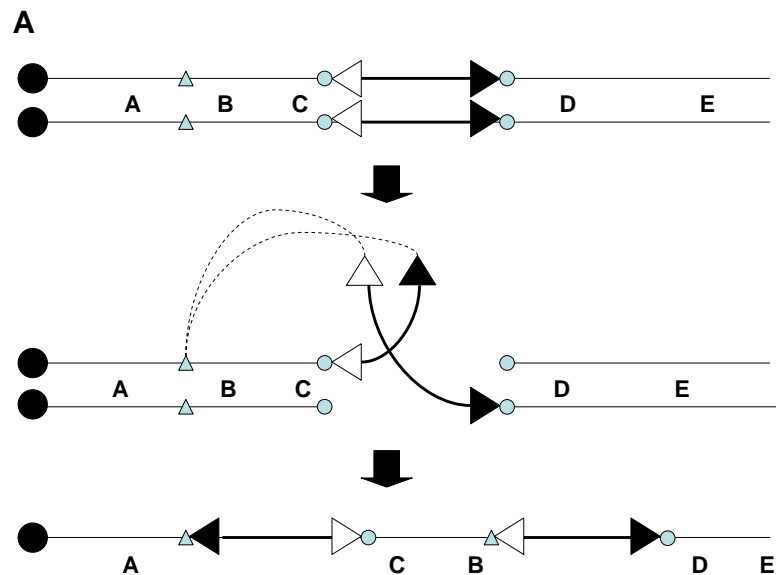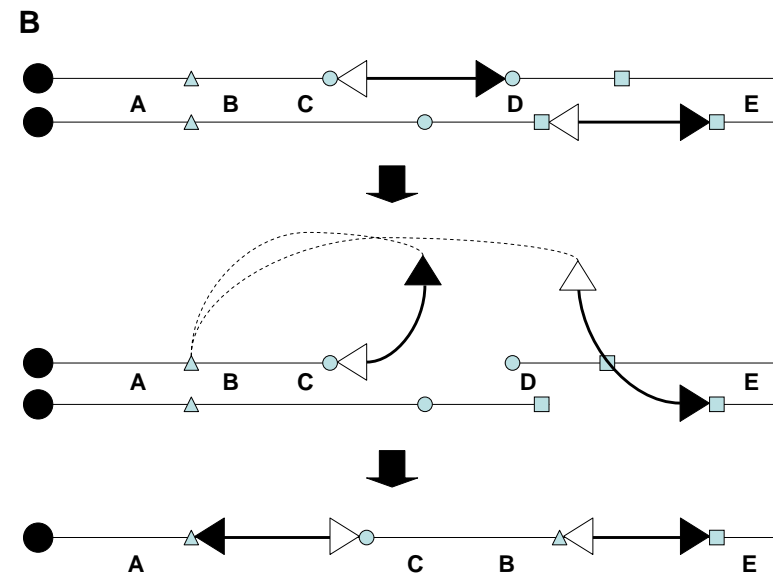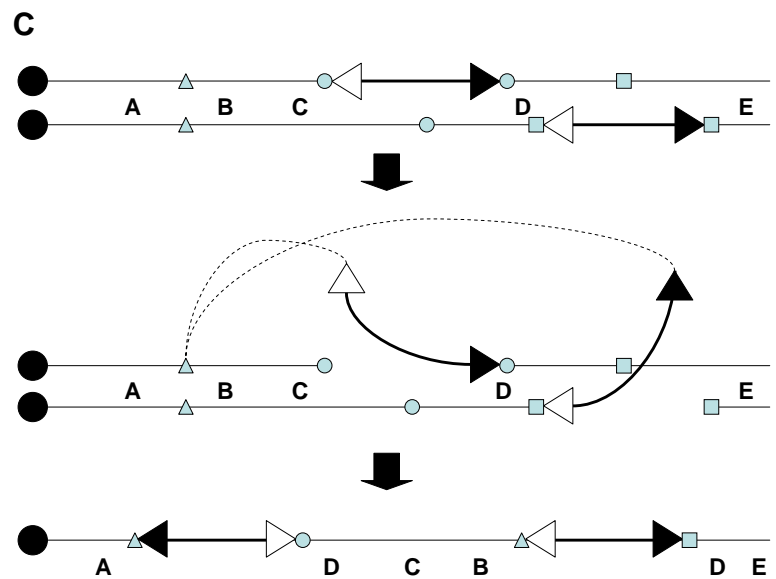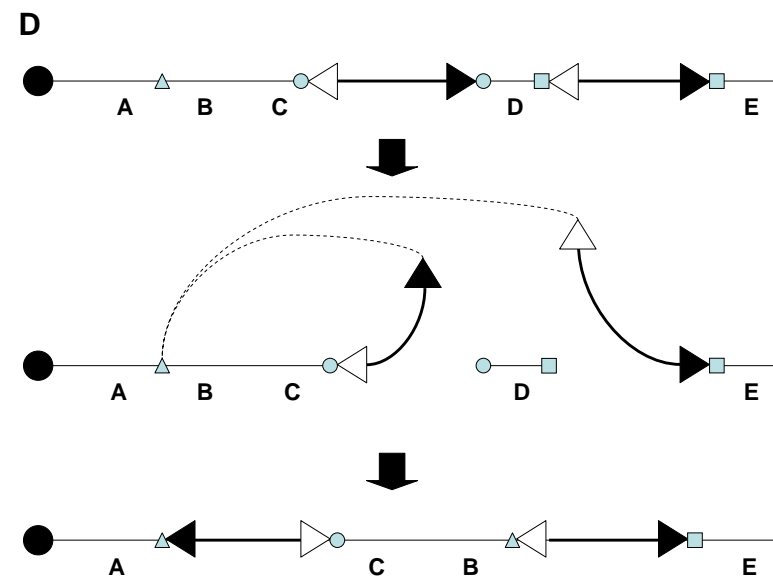

Supplement: Figure S1 — Chromosomal inversions may be generated by transposons when two ends that are not part of the same transposon participate in an aberrant transposition event to a new site [13]–[17]. Target site duplications (TSD) are indicated by ○ or □ (cooperating TE copies) and Δ (new insertion site). (A) The two TE copies are located at the same site of sister chromatids or homologous chromosomes and share the same TSD (○). The result of the aberrant transposition is an inversion (segment BC) flanked by two TE copies. (B) The two TE copies are inserted at separate sites in the two homologous chromosomes and each has its own TSD (indicated by ○ and □). The aberrant transposition event produces an inversion (segment BC) and a deletion (segment D). (C) The two TE copies are arranged as in (B) but two different element ends are involved. The resulting chromosome carries an inversion (segment BC) and a duplication (segment D). (D) The two TE copies are inserted at separate sites on the same chromatid and each has its own TSD (indicated by ○ and □). The resulting chromosome has an inversion (segment BC) and a deletion (segment D). (0.02 MB PDF) [file pone.0007883.s001.pdf]
