# Supplementary material for: The Transposon Galileo Generates Natural Chromosomal Inversions in Drosophila by Ectopic Recombination
Source: PLoS One. 2009 Nov 18;4(11):e7883. doi: 10.1371/journal.pone.0007883 (PMC2775673; doi:10.1371/journal.pone.0007883)
Supplement: Figure S2 — Alignment of gene Mdp sequences in three Drosophila species. The aligned sequences are: positions 50294–51354 from D. buzzatii BAC clone 40C11 (accession number AY900632), positions 6137692–6136590 from D. mojavensis scaffold_6540 and positions 5807143–5806092 from D. virilis scaffold_12855. Yellow boxes indicate exons with the initial methionine and the final stop codon colored in orange and red, respectively. The premature stop codon found in the D. mojavensis sequence is also shown as a red box. Note that there are some parts of the sequence upstream of the coding region that are conserved in the different species suggesting that they may be part of the 5′ UTR or the regulatory regions of the gene. A putative polyA signal determined only on the basis of sequence conservation in the different species is included in a purple rectangle. The blue bar below the alignment indicates the 763-bp fragment amplified by RT-PCR and sequenced in D. buzzatii with primer pair DF-DR. The protein sequence encoded by the D. buzzatii gene is shown above the alignment. The residues enclosed in a green box correspond to the MADF domain found using InterProScan (http://www.ebi.ac.uk/Tools/InterProScan/). (0.01 MB PDF) [file pone.0007883.s002.pdf]

GENE *Mdp*

```
Dbuz    CGAATTGTTGTCATA---------CAACTGGGAGCAGAGGTCTGGCAACTACATGGGCAG
Dmoj    CCAATTGCTGTCATA---------CAATAGGCAGCACAGATCTGGCAACTACTTGGCCAG
Dvir    CAAAGTTTTGCCAGATATTGTAATTAGCTGACAACACGGATCTGGCAACTACCTAGCCAG
        * ** *  ** ** *          *   *  * **  * ************ * * ***

Dbuz    TCGCTGCCATA-------------------------GTCTGGCAGCGCTGTGCCAG----
Dmoj    TCGCTGCCATACAAGC-------TGTGGGCATATTGGTCTGGCAGCGCTATGTCAG----
Dvir    ATGTTGCCATACAAAACTGTCTGCGTTCACGATTAAGTCTGGCAGCACTGTCCAAGCGCG
          * *******                         ********** ** *  **

                                                  M  E  A  R  S  K
Dbuz    ------------ATAGCCGCCAGACACCA----TATGAGCAATGGAGGCGAGAAGTAAAC
Dmoj    ------------GTTTCTGCCAGACGCCA----TATAAGCAATGGAGGCGAGAACTAAAC
Dvir    TGCGGGCATACATTCATTGCCAGATAACAAGCCTAACAAAAATGGAGTCTCGAAATAATG
                     *    ******   **    **  *  ******* *  *** ***

        Q  Y  F  N  D  W
Dbuz    AATACTTCAATGATTGGGTAATATAATAATAAAATCAT-TTTTAAAGTAAGAAAAGGAAC
Dmoj    AACACTTCAATGATAGGGTAATATAATAATAAAAATATCTTTTTCAATAA----------
Dvir    AATTTCTGAAT---TGGGTAATTTCACAAT-------TGTTTTTAATGAAA---------
        **    * ***    ******* * * ***       * ****  *  **

                                           M  K  A  K  K  R  C  R  R
Dbuz    TTTAAT-TCAAGTAT--A---TA-ATATTCCTAGATGAAAGCCAAGAAACGTTGTCGGCG
Dmoj    -TTGAT-TTAAACATTAA---TACATATTTGCAGGTAAGCGCCAAGAAACGCTGTCGGAG
Dvir    -TTGTTCTTAACCATATAAAGTACACTCCAGGAGACAAGCGAAACCAAACGTCCGCGCAG
         **  * * **  **  *   ** *       **   *  *  *  *****    **  *

          S  R  K  I  E  K  E  W  T  P  D  D  V  H  L  L  I  R  L  V
Dbuz    ATCGCGAAAAATCGAAAAGGAGTGGACGCCAGACGATGTTCATTTGCTCATCCGACTCGT
Dmoj    ATCGCGAAAAATCGAACAGGAGTGGAAACCAGATGATGTCCGTTTGCTCATCCGGCTCGT
Dvir    GATACGAAAAATCGAACGGGAGTGGTTACCAAACGATGTCCGGTTGCTAATTCACCTGGT
            ************  *******   *** * ***** *  ***** ** *  ** **

          G  Q  R  E  L  L  W  D  P  S  N  A  N  H  K  D  G  K  L  R
Dbuz    GGGACAACGGGAATTACTGTGGGATCCAAGCAACGCGAATCATAAAGATGGCAAATTGCG
Dmoj    GGGACAGCGGAAATTGCTATGGGATCCAAGCAATCCGAATCACAAGGATAGCAAATCGCG
Dvir    TGAGCAGCGCAAAGTCTTGTGGGATCTCAGCAAATCGAACCACAAAGATAGCAAGTTGCG
         *  ** **  ** *  * *******  *****  **** ** ** *** **** * ***

          E  E  A  F  K  I  I  A  S  T  L  D  R  T  L  T  D  C  K  A
Dbuz    TGAGGAAGCATTTAAAATTATAGCCAGCACACTAGATCGCACGCTGACCGACTGCAAGGC
Dmoj    CGAGCAAGCATTTCAAATGATAGCCAGCAAACTGGATCGCACCCTGGCCGACTGTAAGGC
Dvir    GGAGAACACATTTCAATCTATTGCCAAAACTCTGGATCGCACAACGCCCGACTGCAAGGC
         *** *  ***** **   ** ****  *  ** ********   * ******* *****

          K  W  D  N  L  R  A  Q  Y  R  S  Y  Q  A  K  E  S  Q  N  I
Dbuz    TAAATGGGACAATTTGCGAGCTCAATACAGAAGCTATCAGGCAAAGGAGAGCCAAAATAT
Dmoj    CAAGTGGGACAATCTGCGAGCCCAATATAGAAGCTATCAGGCGAAGGCGACCCAAAACAT
Dvir    CAAGTGGGATAATCTGCGTAACCAATACAGAAGCTACCAGGCCAAGGCGAATCATAACAT
         ** ***** *** ****    ***** ******** ***** **** **  ** ** **

          E  I  K  W  Q  Y  Y  E  S  L  Q  F  L  H  Q  V  C  D  P  R
Dbuz    TGAGATCAAATGGCAATACTACGAGTCATTACAATTTCTGCATCAAGTCTGCGATCCACG
Dmoj    TGAGATTAAATGGCAATACTTTGAGCCTCTAAGCTTTTTGCATGAAGTCTGCGAGCCACG
Dvir    TGAAGTCAAATGGCAATACTTTGAATCATTAAGTTTTCTGCAAAAAGTCTGCGAGCCTCG
        ***  * *************  **  *  **   *** ****  ********** ** **

          K  S  K  G  Y  S
Dbuz    CAAGTCTAAGGGTTATTCTGTAAGTTATATAGTTATAATAAGTAAAT-AA-------TAT
Dmoj    CAAGTCCAAAGCCAATTCTGTAAGTCA------CCAAACACATAAAT-AAAAATACTTAT
Dvir    CAAGTCCAAAGCCAATTCGGTGGGTTA--------GCACACGTATATCAATAATACTT--
        ****** ** *   **** **  ** *          * *  ** ** **       *
```

```
                                                                F  E  L
Dbuz   G-ATATAGATATATGTTTAT-ATTAATTCCA-------TTTCATTCACAGTTCGAGTTG-
Dmoj   GTGTATATATATATGTACATAATTAATATGATATCAATTTTCAGTTACAGCTCGAACCG-
Dvir   ---TATAGAACTCTTTTTTT-TTTAATGAAA-------TTCCAGATAAACCTGGCTTCGA
          **** *  * * *   *  *****   *       ** **   * *  * *    *

                N  D  E  V  S  I  K  R  A  P  S  Q           P  L
Dbuz   --------AATGATGAGGTTTCTATCAAACGTGCACCTTCACAA---------CCTTTAA
Dmoj   --------AATGATGAGGTTTCTATCAAGCGCTCACCCTGACTG---------TCATTGA
Dvir   ATAACATCAATGATACAATATCTATCAATCGTCCGCTTTCACAGAGCCATATGCATTTCA
               ******    * ******** **  * *  * **              ** *

       M  E  D  L  D  T  C  S  T  L  L  S  F  A  T  K
Dbuz   TGGAAGATCTGGACACCTGTTCCACTCTGCTCTCCTTTGCTACGAAA-------------
Dmoj   TGGAAGAGGTGGACACCTGCTCCACTATGTTCTCCTTGGCTGCGAAT-------------
Dvir   TGGATGATTTGGACACCTGCTCCAGTTCGTTTTCCACGTCCACGCATGGAGTTAAGACTA
       **** **  ********** **** *  * * ***    *  ** *

                         A  H  E  E  Q  R  R
Dbuz   -----------------GCACATGAAGAGCAAAGACGA---------------------T
Dmoj   -----------------GCTCCAGAACTGCACGAAGGA---------------------T
Dvir   AAGAGGAGCTGAACAAATCGCCGGAGTTGAATGATCGAAGCAAATCGGATAAAACGGATT
                         * *  **   * *     **                     *

       S  L  T  V  R  E  P  N  P  H  S  H  H  I  F  G  E  F  V  A
Dbuz   CTTTAACAGTGAGGGAGCCTAATCCCCATTCCCATCACATCTTTGGCGAGTTTGTGGCCG
Dmoj   CTTTAGCTGTAAGGGAGCCAAACACACATTCTCACCAAATCTTTGGCGATTTTGTAGCCG
Dvir   CTCCGCCCGAGACAAATCATAATCATAATTCCCCCCAAATTTTTGGCGATTTTGTGGCAG
       **    * *  *   * *  **     **** *  ** ** ******** ***** ** *

       D  E  M  S  R  L  Q  P  H  I  A  D  K  L  K  R  N  I  L  K
Dbuz   ACGAAATGAGCCGCCTGCAACCCCATATAGCCGATAAGCTAAAGCGAAATATCTTGAAAC
Dmoj   ACGAAATGCGCCGCCTGCCGCCCCATATAGCTGATGAGCTAAAGCGAAATATCCTAAAAC
Dvir   ATCAAATGCGCCAGCTTAAACCACAAATAGCTGATCAACTGAAGAGAAATATTTTGAAAC
       *  ***** ***  **    ** ** ***** *** * ** *** *******  * ****

       L  I  V  E  A  L  D  K     Q Stop
Dbuz   TAATTGTCGAGGCATTGGACAAA---CAATAATTG--------GAATATACTAACTTGGT
Dmoj   TGATTGTCGAGGCATTGGACAGA---CAATAGTTGAAAAATATAAATATATTATCCTAAC
Dvir   TAGTTGTCGAAGCCTTAGATAAAAATAAATAA-------------ATGTA-TGCCTTA--
       ******* ** ** ** ** * *    ****              ** ** *  * *

Dbuz   TGAATTAA--TTGTTACTCATTATATTCCCAAACTGTTCTGTATTTAGGCAATCTTAGTA
Dmoj   TGAATTTATTTTATTACCAATTTTGATTTC--ATTAGTCTGTATTTCAGTATTTTTCGCA
Dvir   ------------------------------------------------------------

Dbuz   TCAAGCACAAAATTATACAACTAGAATTATTAATTATTTCA-------------------
Dmoj   ATCTGTACAGAAATATACTAATAAAACTATTAATCAATGCATTGTTAACATACTGAATTT
Dvir   ------------------------------------------------------------

       polyA signal
Dbuz   AATATAA---TTATTTAAGTTAATATATATATATGT---------TAAAATTT
Dmoj   AATATAATATTTATTTTAATTGATAAATGTAAATGTTCTATGAAATGAAATTC
Dvir   AATATAA------GTTAAAATAATATGTCCAGATA----------TAAGATTC
       *******       ** *  * ***  *  * **           * * ***
```
