# Supplementary material for: The Transposon Galileo Generates Natural Chromosomal Inversions in Drosophila by Ectopic Recombination
Source: PLoS One. 2009 Nov 18;4(11):e7883. doi: 10.1371/journal.pone.0007883 (PMC2775673; doi:10.1371/journal.pone.0007883)
Supplement: Figure S3 — Alignment of gene Dlh sequences in two Drosophila species. The aligned sequences are: positions 52175–55219 from D. buzzatii BAC clone 40C11 (accession number AY900632) and positions 6136143–6133352 from D. mojavensis scaffold_6540. This gene could not be found in the D. virilis genome sequence. Yellow boxes indicate exons with the initial methionine and the final stop codon colored in orange and red, respectively. Enclosed in a purple rectangle is the codon in the second exon of the gene that becomes a polymorphic premature stop codon in lines j-9 and jz3-1 by changing from TCA to TAA. No further upstream non-coding sequence could be included in the alignment because of the presence of a polymorphic GalileoN insertion in the st-1 line, from which the D. buzzatii BAC clone is derived. Bars below the alignment in different shades of blue indicate the three overlapping fragments amplified by RT-PCR and sequenced in D. buzzatii with primer pairs CF-CR (278 bp), CF-RT1R (609 bp) and RT2F-RT2R (1,011 bp). The protein sequence encoded by the D. buzzatii gene is shown above the alignment. The residues enclosed in a dark green box correspond to a SNF2-related or a DEAD-like helicase N-terminal domain and the aminoacids in a light green box correspond to a DNA/RNA helicase C-terminal domain. The protein domains have been analyzed using InterProScan ((http://www.ebi.ac.uk/Tools/InterProScan/). (0.02 MB PDF) [file pone.0007883.s003.pdf]

GENE *Dlh*

```
Dbuz      CGCTTGTGAATAAAAGATCGAAAGTAATA----------TATAATACATATATGTACATA
Dmoj      CGCTTGTGCGTCAGAGCTCCAATGTGAAATTCTTGCTAGTAGAATACATATATGTATAAT
          ********  * * ** ** ** ** * *          ** ************** *

Dbuz      CATACGTCAGTAGTTCTTG--------------AGTAACCACCCGGAACATATTACTCTA
Dmoj      GATAAAGCATTAGTTTTTGTGTATTTCCCCAACAGTAAGCATCCGGATCTTTTTTCGCAA
           ***   ** ***** ***              ***** ** ***** * * ** * * *

                                     M  P  V  G  V  S  V  P  L  S  I
Dbuz      CTGAACGTATTTTAAACACAATCAAAATGCCCGTTGGTGTGTCTGTTCCGTTATCTATAA
Dmoj      CGTAACTAATTTTATACTCACCCAAAATG------------------CAGTTAAATATTA
          *  ***  ****** ** **  *******                  * ****  *** *

          N  S  S  E
Dbuz      ATTCGAGCGAGGTAAGAGTTTGAAATATATGTTATTAAAGATTGTTTTTAAAAAAAATTT
Dmoj      GTTGCAGCGAGGTAAGTGTTTAAAAAAACTATTAAACAAAATT-TTATTACAAATATTTA
           **  *********** **** *** *  * ***   ** *** ** *** *** * **

                      E  L  M  D  E  L  A  R  F  S  G  I  N  T  V  K
Dbuz      ACTCCTTTTAGGAACTGATGGACGAGTTGGCCAGATTTTCGGGCATAAATACTGTTAAGC
Dmoj      ATTACGTATAGGAACTCACGGACGAATTGGCCAGATTTTCAGGCAGATATACGGTACAGC
          * * * * ******** * ****** ************** **** * **** **  ***

          P  E  T  I  S  G  L  K  V  K  L  E  Y  H  Q  A  Q  A  L  G
Dbuz      CGGAAACTATATCAGGTCTCAAGGTGAAACTAGAATATCATCAAGCCCAAGCGCTCGGAT
Dmoj      CCGATAGTTTATCAAACTTCGATGTGCATCTCGAGAATCATCAAATAGAAGCTCTCCAGT
          * ** * * *****    ** * *** * ** **  ********    **** ***   *

          W  M  H  M  C  E  M  L  E  P  F  G  G  I  L  A  D  D  T  G
Dbuz      GGATGCACATGTGTGAGATGCTGGAACCATTTGGTGGCATTCTGGCCGATGACACAGGAT
Dmoj      GGATAAACCTGTGTGAGAATATGGAACCATTTGGTGGTATCCTGGCCGATGACAAAGGAT
          ****  ** *********   **************** ** ************* *****

          L  G  S  T  W  T  I  I  A  W  L  L  I  Q  K  L  Q  N  Q  E
Dbuz      TGGGCAGTACATGGACAATTATTGCCTGGCTGCTCATACAGAAACTGCAAAATCAGGAAG
Dmoj      TAGGCAGTACGTGTACTATAATTTGCTGGCTACTCCTCAAAAAACTGGAGAATAGGCACT
          * ******** ** ** ** ***  ****** *** *  * ****** * ***  * *

          D  T  H  S
Dbuz      ACACACACTCATGTGAGTTACGCCCTGATCGAAACACTGATATTGAGAATTTCTCCTCTT
Dmoj      CCACACACTCGAGTGAGTAAAGCACTAA-------GCTAATAT-----------------
           *********  ****** * ** ** *        ** ****

Dbuz      GTCTATATTTATTGAAACTATAAA-TAAATATAGACAAGAGGAGAAACTCTCAATATCAA
Dmoj      -------TTTATTAAAAATGCGAATTATGTGTAAACTGAAAG------------------
                 ****** *** *   ** **  * ** **   * *

Dbuz      TCTCTTTTTCTCTCTCTCTCAATTAAAAAAAAAAAAAAAAACAATTAAAAATTCTCTCGA
Dmoj      -------------------CAGATAAGCAAAAGAAGAAGGCGTGGCAGACAT--------
                             **  ***  **** ** **        * * **

Dbuz      CTCGAAGTCGACCGAGCAGTATTACTTGTTCTGAATTTGAATTATCCATG---TACATAC
Dmoj      -----GGTAGACAATAAAATAT---ATATTCTTGATCAGCATGATCCGCAAACTATGTGT
                ** ***     * ***    * ****  **  * ** ****      **  *

Dbuz      ATACAAATGGGGTATACTAGCTTTATGCATATATGAATATGTAAAAATCATATAT-ATTT
Dmoj      AGACCACTGAG-----------------ATAAATACATATTTCAAATTCATTTATCAGTC
          * ** * ** *                 *** **  **** * *** **** *** * *
```

```
Dbuz        TTATCAGAATG--ACTTGCTGAATCTTTATATCTCGACTAGAGCTTTCTTACTTGTTTAA
Dmoj        CCATACAAACAATATTTAAAAAATGTTTATGTTTTG-----------------TATTTAA
              **   **    * **    *** ***** * * *                 * *****

Dbuz        GCGTAGCTTAGTTAACATTTGTGATGAAAATATTAAATTTATTGTTGGAGCTTTTGTAAT
Dmoj        --------TACTTAGTA-TTGCGTTGAA-----CAAATTGATAGTT----CATCTGCAA-
                    ** ***  * *** * ****      ***** ** ***    * * ** **

Dbuz        AAATAAACAGACATGGCATATTTAGGGTTTTGGATACTCATACTTGAGATTTGTTCACAC
Dmoj        -----------------------------CCGGACATTCATAC---ATATATGTACATAT
                                           *** * ******   * ** *** ** *

Dbuz        TCATATATGGACAAATACTGA--TATGAGTGGCGTCAGCACGTCTTAAAACTTGATTACG
Dmoj        ACATATGT------ATGCTCAAGTGTGAGTGG----------------------------
             ***** *      ** ** *  * *******

Dbuz        AAATTCCATCCAAACGAAATAGTTCTCAA----TAAAAATTTA--AAATGTATACTAAAG
Dmoj        ---------CCATGCGCAACGGTCTGCAATTTTTAGCAACTTATCAACTATTTATTATAT
                     ***  ** **  **   ***    **  ** ***  ** * * ** ** *

Dbuz        AACTAAAAATGTTTCTTAATTAATTATAACAAGTAATTAATTAATTAATGTAATAATTAA
Dmoj        ACATACATAT-----TTGATAAGTTCCTACATATTTCTGACCATTTCGCATATCCACTCA
            *  ** * **     ** ** * **   ***  *   * *  * **    **   * * *

Dbuz        ATATAACAATTAATAACGTTGGAGTCGGAAGTTACCGATTGTATAAGACTCATTCACAGC
Dmoj        ATATA--TATACATAACTATATA----------------TGTATAAAAGATATT-AAAAC
            *****   **  *****  *  *                ******* *   *** * * *

Dbuz        CCGGACGTTGTCATATGTATTTAATTTCGAATTTAATAAGTGAACGGCAAGCAGCAAGCG
Dmoj        AACAACACTTTTACTTGTTGTGATTCTCATATTCCATGTGTAGAT-----------AATT
                **  * * *  ***  * * * **  ***  **  **  *            *

Dbuz        CATTTTATATACATACACCACTTT-----TGTGTTAGCTACCGA-ATCTGATAATAAGAA
Dmoj        CAATTCGAGAACAAGCAAAACTGCTCAAATCGGTTGGCAGATGATGTTTGTTTATA----
            ** **     ***  **  ***       *  *** **    **  * ** * ***

Dbuz        AGTGGCGCTGGTATTAAGTTCTATACAAGCTACACTGAGGTAAATGGGTTCGATTCCCGC
Dmoj        -------TTCGTATGACTTTGTGTACTTACGACTTTG-GATGATTGAAT---------GC
                    * **** *  ** * ***   * **  ** * * * **  *         **

Dbuz        AGAAAAAAAAATTTTCTTTTTCCAAAAATAT----GGAACTTTTTAATTTGTATATTTAT
Dmoj        AGAAGCATAAAATAT-------CAAAGGTGTTAAGGGTATTTTCAAAGTCAAAAAGTT--
            ****  * *** * *       ****  * *    ** * ***  ** *   * * **

Dbuz        ATTTATTATAAAATGTATGTCAGCTAAAAAATTTATAATATTTATTTATATTTATAAACA
Dmoj        -CCTA-CATACATTGTATTGTAGGGTAGACTTTTAT-ATAAAGATAAACATACATAAACA
               **  *** * *****   **   * *  ***** ***   **  * **  *******

Dbuz        TATGCGTATGAATTGTATATTTAGGCACTAGA--GACTGTGTTTCCGATTTGCCCAGGTC
Dmoj        T----TTAAAAAATATTTATTTAATAACTAGAAGAATTCTTCTTCC--------------
            *     **  ** * * ******   ******   * * *  ****

Dbuz        CTAGTGGGTGGATCGACAAAATTTGTAC-ACTTTATAATTCGGGAGCATACATGAGTATG
Dmoj        ---------------------CTTATACTACTTCGTA-----------------------
                                  ** *** ****  **
```

```
                                                    C  Y  G  G  E  T
Dbuz    TGGTCCAAATTGCAAGTCTCTGGCTCTAATGAATTTG-TTTTAGGTTATGGTGGCGAAAC
Dmoj    -------------------CTG---TTACTTAATTTGTTTTTAGACTATGCTGGCGAAAC
                           ***    ** * ****** ******  **** *********

          L  I  V  C  P  K  S  I  L  S  K  W  V  D  D  I  Q  A  V  A
Dbuz    ACTGATTGTGTGTCCTAAAAGTATACTGAGCAAGTGGGTGGATGATATTCAAGCCGTGGC
Dmoj    TCTGATTGTATGTCCAAAAGCTATCCTGACCCAGTGGGTAGATGAGATTAGAGCTGTGGC
         ******** ***** ***  *** **** * ******* ***** ***  *** *****

          G  D  Q  L  K  V  Y  M  Y  Y  G  K  N  R  D  I  S  E  I  S
Dbuz    AGGTGATCAGCTAAAGGTCTATATGTACTATGGAAAAAATCGAGACATCAGTGAGATCTC
Dmoj    GGGTGATCAGCTTAGGGTCTATGAGTACATCGGAAAAAATCGCGACATCAACAACATTTC
         *********** * *******  ****   *********** *******   *  ** **

          S  A  D  I  V  I  T  S  Y  T  V  V  K  Y  D  W  N  N  D  R
Dbuz    AAGCGCAGACATTGTGATCACATCCTATACTGTTGTGAAATACGATTGGAATAACGATAG
Dmoj    TAACGCTGACATTGTGATCACCACCTATAGAGTTGTAACGTATGATGCGAAGAAAGAAAC
         * *** **************  ******  ***** *  ** ***  *** ** ** * 

          N  L  L  N  P  N  W  R  R  V  I  L  D  E  A  Q  A  I  R  N
Dbuz    GAATCTCTTGAATCCCAATTGGCGTCGAGTCATATTGGATGAGGCCCAAGCCATAAGAAA
Dmoj    GAATCCGCTGAATCTCAAATGGCGTCGCCTCATTTTGGATGAGGCTCAAGCCATAAGAAA
        *****   ****** *** ********  **** *********** **************

          I  Q  T  E  R  S  K  A  I  S  A  L  K  S  R  F  R  W  A  V
Dbuz    TATCCAAACAGAACGTTCAAAAGCAATCAGCGCTCTGAAGAGCCGTTTTCGATGGGCTGT
Dmoj    TACCAATGCGGAATGTTCAAAAGCAATCTGCGCTCTGAATAGCCATTTTCGATGGGCTGT
        ** * *  * *** ************** ********** **** ***************

          T  G  S  P  I  Q  N  S  T  H  D  F  M  A  L  L  G  F  L  F
Dbuz    GACTGGATCTCCAATACAAAATAGTACACATGATTTTATGGCATTGCTCGGCTTTCTGTT
Dmoj    GACTGGAGATCCAATCCAGAATAACGAAAAAGATTTTATGGCATTGCTCGGCTTTCTGTG
        *******  ****** ** ****    * * **************************** 

          R  G  P  I  K  M  D  R  C  L  C  L  R  R  T  I  S  D  V  Q
Dbuz    CCGAGGACCCATTAAGATGGATAGATGCTTGTGCTTAAGGCGCACCATATCTGATGTTCA
Dmoj    CAGAGGACAGTTAAGGAAGGATAAACGTTTGTGCTTAAGGCGTACAATGTCTGATGTTCA
        * ******   * * ** ***** * * ************** ** ** ***********

          T  T  G  Q  L  Q  N  F  P  Q  L  I  P  K  E        I  K  I
Dbuz    AACAACTGGACAGCTTCAGAATTTTCCACAGCTAATACCCAAGGAA------ATTAAAAT
Dmoj    CTCAACTGAACAGATTCAGAAATTTCCAACTTTA------AATGAATTTTCCATTGAAGT
          ****** **** ******* ******    **      ** ***      *** ** *

          E  L  N  E  Q  E  R  I  L  Y  N  K  V  R  S  K  L  D  L  C
Dbuz    AGAACTGAATGAACAAGAAAGGATATTGTATAACAAAGTTCGTAGCAAATTGGATCTATG
Dmoj    AGAACTTAATGTACAAGAAAGAATATTGTATGACAAAGTTCGTAACAAACTGGATCGACA
        ****** **** ********* ********* ************ **** ****** *  

          V  E  T  R  T  M  I  H  L  V  R  T  Q  R  L  Q  Q  L  C  S
Dbuz    TGTCGAGACGCGCACCATGATACACTTAGTGCGGACGCAACGACTTCAACAGCTGTGCTC
Dmoj    AGTCCAGAGGCGCTCCATGATACACTTGGTGCGGGTGCAACGCCTTCAACAGCTATGCTC
         *** *** **** ************* ******  ****** *********** *****

          H  P  Y  L  I  R  S
Dbuz    TCATCCATATCTAATCCGATCGGTAAGTACTAGAATCAGAATCAATAAAGATCACTGTAA
Dmoj    TCATCCATATCTAATCCGATCGGTAAGTTTCAGAACCTGTGTCTATGAATCTAGACACAA
        ****************************   **** * *  ** ** **  *      **

                                        L  N  I  I  D  Q  N  E  Y  E
Dbuz    TTATTAATATTTTTATTTCTTTGTAATATAGCTCAACATAATTGATCAGAATGAATACGA
Dmoj    TTAAATATATTTTTATTTTATTATGATACAGATGGGCATAATTAAACGGGAGCAATACGA
        ***   ************  ** * *** ** *   ******* * * * *  *******

          G  E  S  T  F  D  C  S  D  T  S  D  E  N  V  L  S  L  R  N
Dbuz    AGGCGAAAGCACTTTCGACTGCAGCGACACCTCAGATGAAAATGTCCTGAGCCTGAGAAA
Dmoj    AGGCGAAGGATATTTCGACTACAGTAACACATCTGATGATAATGTTCTGAGCCTAAGGAA
        ******* *   ******** ***  **** ** ***** ***** ******** ** **
```

```
          P  I  F  H  R  N  T  P  S  T  K  I  S  R  A  I  E  I  I  N
Dbuz    TCCCATTTTTCACCGGAACACTCCTTCAACAAAAATTAGTCGCGCTATTGAGATAATCAA
Dmoj    TCCCATCTTTGACCGGGCCTCTCCTTCAACGAAAATTCTTAAAGCTTCTGAGATAATTGA
        ****** *** *****  * ********** ******  *   ***  *********  *

          E  H  V  L  L  E  K  K  K  I  I  V  V  S  Q  S  V  A  L  L
Dbuz    TGAACACGTACTACTAGAGAAAAAGAAAATCATTGTAGTGTCGCAATCGGTAGCACTGCT
Dmoj    TCGATGCATACTGCAAAATAAGGAGAAAATCATTGTAGTATCGCACTGGAAAGAACTGCT
        *  *  * **** * * * **  **************** ***** * *  ** ******

          E  I  L  N  K  H  L  Y  K  D  S  T  R  Q  L  K  I  M  T  L
Dbuz    GGAGATATTGAACAAGCATTTATATAAAGACAGCACACGACAACTAAAGATTATGACACT
Dmoj    GGAAATATTGAAAGAATATTTAAATACCGGCAGC---AGAGAACTAAGAATTATGACGTT
        *** ********  *  ***** ***  * ****    ** ******  ********  *

          T  G  R  T  P  Q  H  K  I  G  E  L  I  S  D  F  N  E  S  V
Dbuz    GACTGGCAGGACTCCACAGCATAAAATCGGAGAATTAATTAGCGATTTTAACGAATCTGT
Dmoj    AACTAGCAGTAATACGCAGGATACAATCAAA------------AATTTTAACGAATCTGA
         *** **** * * * *** *** ****  *             ***************

          K  P  C  I  L  L  I  S  L  K  L  A  E  T  G  L  N  L  N  G
Dbuz    TAAACCTTGCATTCTGCTGATCTCGTTGAAATTGGCAGAAACTGGTTTAAATTTGAATGG
Dmoj    TGAGCCTTGCGTTCTGCTCCTCACTTTGGGATTAGCGAAATCTGGTTTAAATTTATATGG
        * * ****** *******  ** * ***  *** **  ** *************  ****

          A  K  Y  L  L  F  M  D  L  H  W  N  P  H  L  E  P  Q  S  A
Dbuz    AGCGAAATATTTGTTGTTTATGGATTTGCACTGGAATCCACATCTTGAGCCGCAAAGTGC
Dmoj    AGCCAATTCTTTGTTGGTCATGGATTCTCACTGGAATCCGCATCTGAAACCACAAAGCGC
        *** ** * ******* * *******  *********** *****  * ** ***** **

          I  H  R  L  G  Q  Q  N  E  N  V  I  V  F  Q  F  V  C  K  D
Dbuz    CATCCATCGCCTGGGTCAACAAAATGAAAATGTCATCGTCTTTCAGTTCGTGTGCAAGGA
Dmoj    CATCTATCGGATGGACCAGCGAAACAGAAATGTTTCCGTTTATCAGCTCGTGTGCAAGGA
        **** ****  ***  ** * ***   ******   *** * **** *************

          T  V  D  D  H  I  Q  Q  V  Q  Q  T  K  L  S  L  A  F  Q  H
Dbuz    TACGGTGGACGACCACATTCAACAGGTGCAGCAGACCAAATTGAGCTTAGCTTTTCAACA
Dmoj    TACGGTGGATGGCACAATTCAACAGGTGCAGCAAAATAAGTTGAATTTAGCCTTTCAAGT
        ********* * *   ***************** *  ** ****  ***** ******

          F  K  K  Y  T  I  S  K  V  R  E  V  I  N  Y  F  L  N  L  L
Dbuz    TTTCAAAAAGTACACTATTTCAAAGGTTCGAGAAGTTATTAATTATTTTTTGAACCTATT
Dmoj    TCTCAGAGCGAGTGCTATTTCAACAATTCGAAGAGTTATTAAATATTTTCAGAGTCTATG
        * *** *  *    *********   *****  ********* ******  **  ****

          E Stop
Dbuz    GGAATAACTCTTATATATGTAATGCTGTTTATTATTAA-ATATTGA--------------
Dmoj    GCTGTAATTTTTAT-TTTGTAGAATTGTAAATTGTCAATATATCAAACTAGAAATATTAT
        *   *** * **** * ****    ***  *** * ** ****  *

Dbuz    ----TTGACTCTTGTGCAACATTGAT-----ATTTATTTATTTATTTA------------
Dmoj    ATCTTTATTTCTTGTTTGATTTTAATAGCAGAGCTGTTAGTTAACTTAACACGTAACGGT
            **   ******   *  ** **     *  * **  ** * ***

Dbuz    ---------------------------------------------------------TTT
Dmoj    AAGAAATATAAGTGACTGTTCGCCCGCGCCAGACCACTGACCAGAGAACACAAACGCTTT
                                                                 ***

Dbuz    AAAAATATTGACATAAC
Dmoj    TAAAATTTCGATGTAAC
         ***** * **  ****
```
