# Supplementary material for: The Transposon Galileo Generates Natural Chromosomal Inversions in Drosophila by Ectopic Recombination
Source: PLoS One. 2009 Nov 18;4(11):e7883. doi: 10.1371/journal.pone.0007883 (PMC2775673; doi:10.1371/journal.pone.0007883)
Supplement: Table S1 — Nucleotide variability in non-inverted chromosomes. N = number of chromosomal lines; m = number of compared nucleotides. (0.05 MB PDF) [file pone.0007883.s005.pdf]

Table S1. Nucleotide variability in non-inverted chromosomes. N = number of chromosomal lines; m= number of compared nucleotides.

			2st (N = 3)		*2j* (N = 5)		ALL (N = 8)	
Sequence	Region	m	S	π	S	π	S	π
Non coding	A	301	1	0.0022	3	0.0053	3	0.0040
	B	398	4	0.0067	5	0.0070	7	0.0069
	C	187	1	0.0036	7	0.0160	7	0.0118
	D	496	11	0.0148	8	0.0089	16	0.0147
	AB	699	5	0.0048	8	0.0066	10	0.0057
	CD	683	12	0.0110	16	0.0105	23	0.0139
	BuT5	182	5	0.0183	2	0.0044	6	0.0132
	ABCD	1382	17	0.0082	23	0.0085	33	0.0097
Coding	A (*CG2046*)	216	2	0.0062	3	0.0083	3	0.0073
	B (*CG10326*)	55	0	0	0	0	0	0
	C (*Dlh)*	178	1	0.0037	6	0.0180	7	0.0148
	D (*Mdp)*	270	1	0.0025	1	0.0022	2	0.0032
	ABCD	719	4	0.0037	10	0.0078	12	0.0071
