# Supplementary material for: The Transposon Galileo Generates Natural Chromosomal Inversions in Drosophila by Ectopic Recombination
Source: PLoS One. 2009 Nov 18;4(11):e7883. doi: 10.1371/journal.pone.0007883 (PMC2775673; doi:10.1371/journal.pone.0007883)
Supplement: Table S2 — Structure and similarities of two novel Drosophila genes: MADF domain protein (Mdp) and DEAD-like helicase (Dlh). NT = nucleotide; AA = amino acid. (0.01 MB PDF) [file pone.0007883.s006.pdf]

Table S2. Structure and similarities of two novel Drosophila genes: *MADF domain protein* (*Mdp*) and *DEAD-like helicase* (*Dlh*).

NT = nucleotide; AA = amino acid.

Gene	Region	*D. buzzatii*	*D. mojavensis*	NT identity (%) Dbuz/Dmoj	AA identity (%) Dbuz/Dmoj	*D. virilis*	NT identity (%) Dbuz/Dvir	AA identity (%) Dbuz/Dvir
Mdp	Exon 1	36	36	91.67	75.00	33	69.70	45.45
	Intron 1	69	62	-	-	60	-	-
	Exon 2	345	345	83.19	80.00	345	71.88	67.83
	Intron 2	74	84	-	-	64	-	-
	Exon 3	270	270	80.37	71.59	348	68.52	53.93
	Total	794	797	-	-	850	-	-
	Coding	651	651	82.49	-	726	70.37	-
	Protein	216	216	-	76.28	241	-	60.93

Gene	Region	*D. buzzatii*	*D. mojavensis*	NT identity (%) Dbuz/Dmoj	AA identity (%) Dbuz/Dmoj
Dlh	Exon 1	45	27	74.07	55.56
	Intron 1	60	59	-	-
	Exon 2	241	241	73.86	63.75
	Intron 2	1143	808	-	-
	Exon 3	632	632	80.99	70.67
	Intron3	69	69	-	-
	Exon4	636	621	73.91	59.22
	Total	2826	2457	-	-
	Coding	1554	1521	76.83	-
	Protein	517	506	-	64.48
