# Supplementary material for: The Transposon Galileo Generates Natural Chromosomal Inversions in Drosophila by Ectopic Recombination
Source: PLoS One. 2009 Nov 18;4(11):e7883. doi: 10.1371/journal.pone.0007883 (PMC2775673; doi:10.1371/journal.pone.0007883)
Supplement: Table S3 — BAC clones used for in situ hybridization. (0.01 MB PDF) [file pone.0007883.s007.pdf]

**Table S3**. BAC clones used for *in situ* hybridization.

| Contig | BAC Clones | Cytological bands | Mapping in $2jz^3$ arrangement |
|---|---|---|---|
| 961 | 6M07 | 2E4d | Distal |
| | 14G17 | 2E4d | Distal |
| | 15L20 | 2E4d / 2F1f | Distal / Proximal |
| | 15P22 | 2E4d / 2F1f | Distal /Proximal |
| | 18L15 | 2E4d / 2F1f | Distal /Proximal |
| | 5E20 | 2F1f | Proximal |
| | 12I04 | 2F1f | Proximal |
| | 20E14 | 2F1f | Proximal |
| 968 | 14J13 | 2E4d | Distal |
| | 42J20 | 2E4d | Distal |
| | 1B04 | 2E4d | Distal |
| | 22N23 | 2E4d / 2F1f | Distal / Proximal |
| | 16A20 | 2E4d / 2F1f | Distal / Proximal |
| | 22M06 | 2E4d / 2F1f | Distal / Proximal |
| | 40C11 | 2E4d / 2F1f | Distal / Proximal |
