# Supplementary material for: The Transposon Galileo Generates Natural Chromosomal Inversions in Drosophila by Ectopic Recombination
Source: PLoS One. 2009 Nov 18;4(11):e7883. doi: 10.1371/journal.pone.0007883 (PMC2775673; doi:10.1371/journal.pone.0007883)
Supplement: Table S4 — Plasmid clones used as probes for in situ hybridization to map the proximal breakpoint of the 2z 3 inversion. (0.01 MB PDF) [file pone.0007883.s008.pdf]

Table S4. Plasmid clones used as probes for *in situ* hybridization to map the proximal breakpoint of the *2z*^{3} inversion.

Plasmid clone [a]	Position in BAC 40C11	Cytological band	Mapping in *2jz*^{3} arrangement
9F01	47261 - 48791	2F1f	Proximal
9A02	49380 - 50856	2F1f	Proximal
8H04	51200 - 52675	2E4d / 2F1f	Distal / Proximal
7A11	52820 - 54267	2E4d	Distal /Repetitive
8D03	54232 - 55640	2E4d	Distal / Repetitive
11E08	55582 - 57042	2E4d	Distal / Repetitive
1C04	57109 - 58595	2E4d	Distal
9E12	58146 - 59694	2E4d	Distal
6H10	59910 - 61340	2E4d	Distal
8B09	61356 - 62868	2E4d	Distal

[a] These clones belong to the shotgun sublibrary constructed to sequence BAC 40C11 [64].
