# Supplementary material for: The Transposon Galileo Generates Natural Chromosomal Inversions in Drosophila by Ectopic Recombination
Source: PLoS One. 2009 Nov 18;4(11):e7883. doi: 10.1371/journal.pone.0007883 (PMC2775673; doi:10.1371/journal.pone.0007883)
Supplement: Table S5 — Sequence of oligonucleotide primers used for PCR amplification. (0.05 MB PDF) [file pone.0007883.s009.pdf]

Table S5. Sequence of oligonucleotide primers used for PCR amplification.

Primer pair	Forward (5’- 3’)	Reverse (5’ – 3’)	Designed from
1F - 1R	TATTCCGGCTCGGACATTAG	TGTGTCTGGAGCTGAAATGC	*CG1193 D. mojavensis*
2F - 2R	ACGCTGAGCAGGAATTCAGT	CCGCAATAGCAATCGAAAAT	*CG14906 D. mojavensis*
3F - 3R	TAGGGGAATGTTTTGGATGG	GCACCATCTCGGATCGTATT	*Adk3 D. mojavensis*
4F - 4R	CGCGTAGCCTCTTCTACACC	TTAAAGAACCCACGCAGGAC	*CG4674 D. mojavensis*
5F - 5R	AGCAGATCAAGCAAATACGC	CGCTCATCGTGCAGGATTAC	*CG1193 - CG2051 D. mojavensis*
6F - 6R	GCAGCGTCAACAAACCATAA	AGCTGGAGCATCATTTCGAT	*CG2051 - CG1100 D. mojavensis*
7F - 7R	ACTCACGATTGCACTTGACG	TTCGCTTTTCCAATGTTTCC	*CG1100 - CG1218 D. mojavensis*
8F - 8R	TATGTAGCCACGCCATTCAC	TAGCTGGCGTACTCAGATGG	*CG1218 - CG2046 D. mojavensis*
9F - 9 R	GAGGCATTCCAATGCAAAGT	GACGCCGGCATAGTATTGTT	*CG2046 - CG10326 D. mojavensis*
10F - 10R	GGGCTGAATGAGAAGGATGT	GAAGACCGAGGAAAAGCTGA	*CG10326 - CG10324 D. mojavensis*
11F - 11R	CCCTTCGAGGCATTTGATAA	GGATTTGCTGTTGCAGGAAT	*CG10324 - CG8977 D. mojavensis*
12F - 12R	ACTAACCCGAAAGCAGGTGA	ACTTGCAGCTTGAGCAGGAT	*CG8977 - CG14906 D. mojavensis*
13F - 13R	AAGAGACGGAACACGAGAAC	CTCGGAGTCAGTTGTTGATA	*CG17836 D. buzzatii*

	Forward (5’- 3’)	Reverse (5’ – 3’)	Designed from
14F - 14R	AAATAACATATATCAGTGCTCGAAG	CACGTGTGTATGATTTACAATGAG	*CG2520 D. buzzatii*
15F - 15R	AATGTCGATTACGCCTCTAC	AATCAGTCTCGTGCCATATT	*CG2520 D. buzzatii*
D F - D R	CCAATGCCTCGACAATTAGT	AGCCGCCAGACACCATAT	*Mdp D. buzzatii*
C F - C R	TTGGTGTGTCTGTTCCGTTA	TGAGTGTGTGTCTTCCTGAT	*Dlh D. buzzatii*
B F - B R	GCAAGGATCCGTTATTCCAA	GCTGTCCACACACTTCCTCA	*CG10326 D. buzzatii*
A F - N R	GACAAGCACGAACAGTTCAC	GGTCCTAAGTGTGCTCTGTT	*CG2046* - λz^3-96 *D. buzzatii*
CF – RT1R	TTGGTGTGTCTGTTCCGTTA	TTGGAGATCCAGTCACAGC	*Dlh D. buzzatii*
RT2F - RT2R	ATCTCAAGCGCAGACATTGT	ATCCTTGCACACGAACTGAA	*Dlh D. buzzatii*
CR-ER	TGAGTGTGTGTCTTCCTGAT	GATCTCTGCATCAGTGCC	*Dlh D. buzzatii*
EF-DR	CAGTTCCGTTACTTTTGATG	AGCCGCCAGACACCATAT	*Mdp D. buzzatii*
